# Cdc5-Dependent Asymmetric Localization of Bfa1 Fine-Tunes Timely Mitotic Exit

**DOI:** 10.1371/journal.pgen.1002450

**Published:** 2012-01-12

**Authors:** Junwon Kim, Guangming Luo, Young Yil Bahk, Kiwon Song

**Affiliations:** 1 Department of Biochemistry, College of Biotechnology and Life Science, Yonsei University, Seoul, Korea; 2 Department of Biotechnology, Konkuk University, Chungju, Korea; National Institute of Diabetes and Digestive and Kidney Diseases, United States of America

## Abstract

In budding yeast, the major regulator of the mitotic exit network (MEN) is Tem1, a GTPase, which is inhibited by the GTPase-activating protein (GAP), Bfa1/Bub2. Asymmetric Bfa1 localization to the bud-directed spindle pole body (SPB) during metaphase also controls mitotic exit, but the molecular mechanism and function of this localization are not well understood, particularly in unperturbed cells. We identified four novel Cdc5 target residues within the Bfa1 C-terminus: ^452^S, ^453^S, ^454^S, and ^559^S. A Bfa1 mutant in which all of these residues had been changed to alanine (Bfa1^4A^) persisted on both SPBs at anaphase and was hypo-phosphorylated, despite retaining its GAP activity for Tem1. A Bfa1 phospho-mimetic mutant in which all of these residues were switched to aspartate (Bfa1^4D^) always localized asymmetrically to the SPB. These observations demonstrate that asymmetric localization of Bfa1 is tightly linked to its Cdc5-dependent phosphorylation, but not to its GAP activity. Consistent with this, in kinase-defective *cdc5-2* cells Bfa1 was not phosphorylated and localized to both SPBs, whereas Bfa1^4D^ was asymmetrically localized. *BFA1^4A^* cells progressed through anaphase normally but displayed delayed mitotic exit in unperturbed cell cycles, while *BFA1^4D^* cells underwent mitotic exit with the same kinetics as wild-type cells. We suggest that Cdc5 induces the asymmetric distribution of Bfa1 to the bud-directed SPB independently of Bfa1 GAP activity at anaphase and that Bfa1 asymmetry fine-tunes the timing of MEN activation in unperturbed cell cycles.

## Author Summary

During mitosis the replicated chromosomes are distributed equally to the daughter cells. Once the chromosomes have segregated properly, a pathway called the mitotic exit network (MEN) becomes activated to complete mitosis. How MEN activation is coordinated with segregation of the chromosomes is currently a focus of interest. In budding yeast, Tem1 initiates MEN activation and Bfa1 negatively regulates Tem1 with Bub2. The polo kinase Cdc5 also activates MEN by directly phosphorylating and inhibiting Bfa1. The spindle pole body (SPB), which corresponds to the mammalian centrosome, acts as a platform for these MEN components. The Bfa1/Bub2 complex localizes to SPBs and regulates the association of Tem1 with the SPBs. When the spindle aligns correctly along the mother-bud axis, Bfa1/Bub2 is restricted to the bud-oriented SPB. Conversely, when the spindle is misaligned, Bfa1/Bub2 is present on both SPBs and mitotic exit is delayed, suggesting that the spatial distribution of Bfa1/Bub2 controls the timing of mitotic exit. In this study, we identified new Cdc5 target phosphorylation residues in Bfa1 that function in its asymmetric distribution on SPBs and showed that the asymmetric Bfa1 distribution was required for timely mitotic exit during unperturbed cell cycle of the budding yeast.

## Introduction

In eukaryotes, mitotic entry is driven by a rise in cyclin-dependent kinase (Cdk) activity, which is required for the formation of a bipolar spindle and chromosome segregation (For a review, see [1]). For cells to subsequently undergo cytokinesis and enter the G1 phase of the next cell cycle, Cdk-mediated phosphorylation events are reversed and Cdk activity declines (For reviews, see [2], [3]). In budding yeast, this transition, called mitotic exit, is triggered by a signaling cascade known as the mitotic exit network (MEN). The MEN activates and releases the Cdc14 phosphatase from the nucleolus, and this phosphatase reverses the phosphorylation of Cdk substrates and inactivates the mitotic Cdks (For a review, see [4]).

The MEN must be tightly regulated for each daughter cell to receive a complete set of chromosomes. When the MEN is prematurely activated in cells undergoing mitosis, genomic instability results [5]. Therefore, the MEN is a crucial target of various checkpoints that keep mitotic Cdk activity high until the daughter chromosomes have segregated properly. The MEN coordinates spindle position and mitotic progression in asymmetrically dividing cells such as budding yeast, where the division plane is predetermined. A pathway called the spindle position checkpoint (SPOC) ensures that the MEN is activated only if the extended mitotic spindle is correctly positioned. When spindles misalign relative to the division plane, mitotic exit is delayed by preventing the MEN activation [6].

The Tem1 GTPase functions to activate the MEN [7]. The MEN signaling cascade is triggered when the two-component GTPase-activating protein (GAP) for Tem1, composed of Bfa1 and Bub2, becomes inactivated. The polo kinase Cdc5 also contributes to MEN activation by directly phosphorylating and inhibiting the GAP activity of Bfa1/Bub2 and/or disrupting its interaction with Tem1 [8], [9]. Impaired Bfa1/Bub2 GAP activity allows mitotic exit in cells that have either mitotic exit defects or activated checkpoints [10]. Consistent with this, Bfa1 remains unphosphorylated when the SPOC prevents mitotic exit [8]. Lte1, which was once suggested to promote mitotic exit as a putative guanine nucleotide exchange factor (GEF) for Tem1, has been reported to contribute to mitotic exit by controlling asymmetric Bfa1 localization and cell polarization [11], [12]. A recent study demonstrated that loading of Tem1 onto the spindle pole bodies (SPBs) is required for activation of the MEN [13]. Thus a misaligned spindle markedly delays mitotic exit in cells with low GAP activity for Tem1 [10]. These recent studies have suggested more complex ways by which MEN is regulated, including localization of MEN components to the SPB, together with the GTPase-switch model for Tem1.

The SPB acts as a scaffold for many MEN components (For a review, see [4]). The association of Tem1 with SPBs depends on Bfa1 and Bub2, which are mutually required for the other's localization to the SPB [14]. The Bfa1/Bub2 complex localizes to SPBs in an asymmetric manner: as the spindle aligns along the mother-bud axis and the dividing nucleus migrates to the bud neck, the complex is exclusively found on the bud-oriented SPB [14], [15]. Conversely, on misoriented spindles that lead to delayed mitotic exit, Bfa1/Bub2 is present on both SPBs. This suggests that the spatial distribution of Bfa1/Bub2 is directly connected to the control of mitotic exit [15], [16]. Consistent with this hypothesis, a Bub2 variant that localizes to both SPBs throughout the cell cycle prevented mitotic exit in certain MEN-impaired mutants [17]. Also a recent quantitative analysis showed that Bfa1 dynamics at the SPBs establishes asymmetry in MEN signaling and regulates MEN activity. Bfa1 associates with both SPBs in a transient fashion, but its binding to the daughter SPB (SPB^d^) is stabilized by cell polarity determinants and their interactions with microtubules [18]. As a consequence, Bfa1 accumulates on the SPB^d^ during metaphase, whereas it disappears from the mother SPB (SPB^m^), thereby establishing Bfa1 asymmetry [18]. When the spindles are improperly positioned, Bfa1 association becomes highly dynamic on both SPBs, which is required for proper SPOC function [19].

Despite the role in the fidelity of mitosis, the molecular details governing the asymmetry of Bfa1/Bub2 positioning have yet to be fully elucidated. Furthermore, the importance of the asymmetry in the unperturbed cell cycle remains unclear. Bfa1 asymmetry is required for recruiting MEN components exclusively to the SPB^d^ during metaphase [18]. Bfa1 reaches its maximum phosphorylation state when it associates preferentially with the SPB^d^, whereas Bfa1 is unphosphorylated and localizes to both SPBs in SPOC-activated cells [8], [16]. We have identified new phosphorylation sites on Bfa1 that function in directing its asymmetric distribution to SPBs. We present evidence that the phosphorylation of these sites by Cdc5 does not inhibit Bfa1 GAP activity, but induces Bfa1 asymmetry and achieves timely MEN activation during unperturbed mitotic progression.

## Results

### Bfa1 localizes to both SPBs in the kinase-defective *cdc5-2* mutant

**Figure 1:**
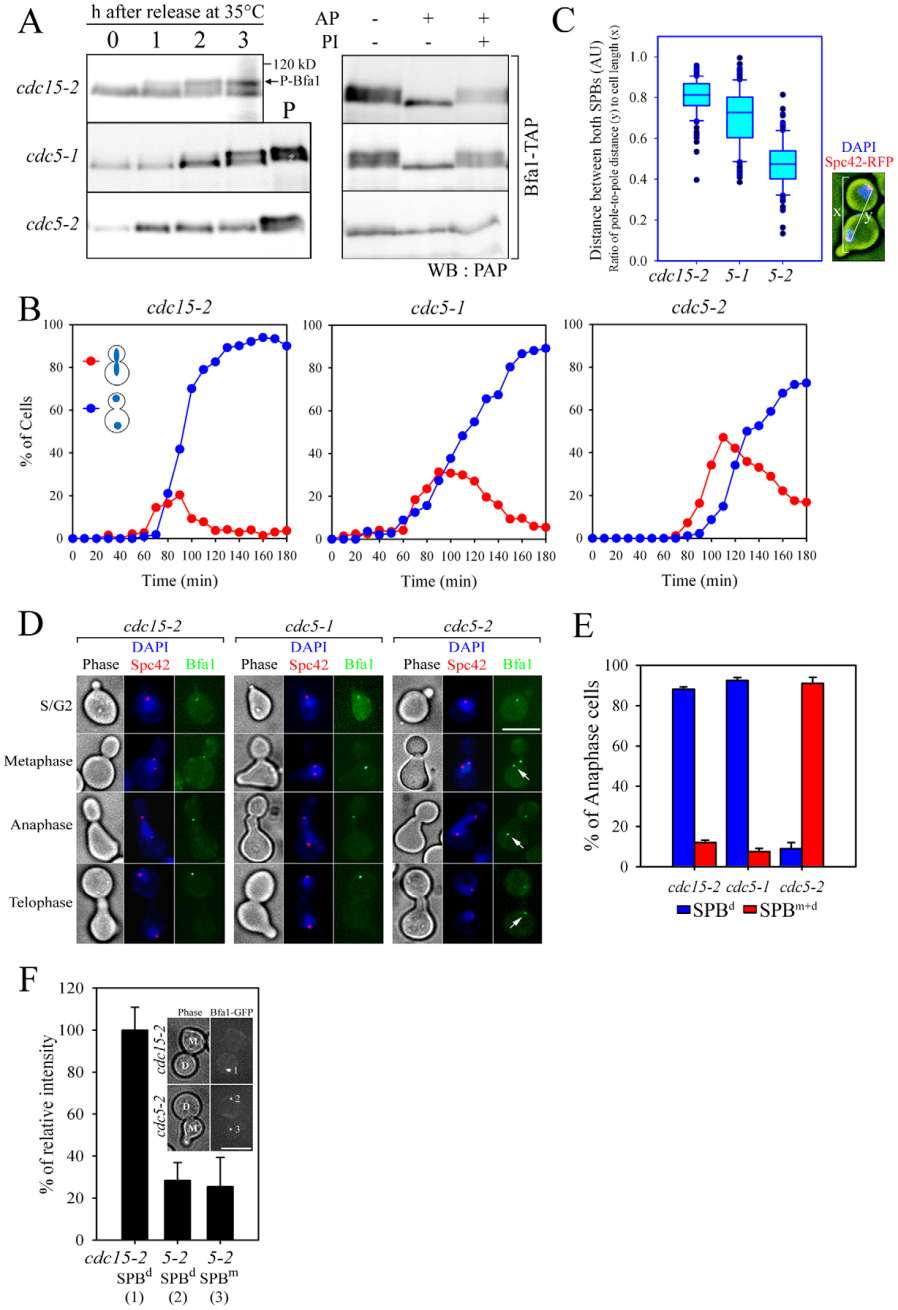
Bfa1 phosphorylation and localization in *cdc5-1* and *cdc5-2* cells. (A) Phosphorylation of Bfa1. *BFA1-TAP* cells with the indicated genotype (YSK1153, 2238, and 1122) were synchronized with α-factor at 25°C, and then released at 35°C. Bfa1 mobility was determined on SDS-PAGE. (Left) Protein extracts were prepared at each indicated time after release. *cdc15-2* cells incubated for 3 h at 35°C provided a positive control (P) for slow migrating forms of Bfa1. (Right) After cell cycle release for 3 h, TAP-tagged Bfa1 was precipitated, and either untreated (−) or treated (+) with alkaline phosphatase (AP) and/or phosphatase inhibitors (PI). (B–E) Phenotypes and Bfa1 localization of *cdc5-1* and *cdc5-2* mutants. Indicated cells with *BFA1-GFPSPC42-RFP* (YSK2545, 2438, and 2442) were released from α-factor arrest at 35°C for 3 h. (B) Cell cycle progression. Cells were collected every 10 min and stained with DAPI. The percentage of indicated cell types was determined (n = 200). (C) Box plots comparing the spindle lengths. The relative spindle length (the ratio of distance between both SPBs (y) to cell length (x) was determined in cells with two segregated nuclei after release for 3 h (n = 119 for *cdc15-2*, 116 for *cdc5-1*, and 287 for *cdc5-2*). The line inside the box indicates the median. Pole-to-pole distance was determined using Spc42-RFP as a SPB marker. AU, arbitrary unit. (D) Localization of Bfa1-GFP to SPBs. Cells were fixed and stained with DAPI. Representative images showing Bfa1-GFP localization during mitosis were taken by fluorescence microscopy. Arrows indicate Bfa1-GFP at the SPB^m^. Note that daughter cells can be clearly distinguished from the mother by the presence of a mating projection. Bar, 5 µm. (E) The quantification of Bfa1 localization. After cell cycle release for 3 h, cells with indicated phenotypes were counted (n = 200). The average of two independent counts is plotted with standard deviations. SPB^d^, anaphase cells with Bfa1 only at the daughter SPB. SPB^m+d^, anaphase cells with Bfa1 at both SPBs. (F) The fluorescence intensity of Bfa1-GFP. After cells with the indicated genotypes (YSK2545 and 2442) were released for 3 h, images were taken by confocal microscopy. GFP fluorescence intensity was quantified as described in Materials and Methods, and plotted relative to the intensity of the SPB^d^ in the *cdc15-2* mutant (n = 23 for *cdc15-2* and 26 for *cdc5-2*). D, the daughter SPB, M, the mother SPB. Bar, 5 µm.

Bfa1 is a cell cycle-regulated phosphoprotein [8] that forms a complex with Bub2 and negatively controls the activation of Tem1, a key upstream regulator of mitotic exit [8], [9]. Cdc5 polo kinase phosphorylates Bfa1 during mitosis to down-regulate Bfa1/Bub2, and thus activates mitotic exit [8], [9]. In *cdc15-2* cells, Bfa1 becomes phosphorylated by Cdc5 and Tem1 is activated, but mitotic exit is not permitted since Cdc15 acts downstream of Tem1 in the MEN [7]. Thus, we used the *cdc15-2* mutant, which contains wild-type *CDC5*, as a control for Bfa1 phosphorylation and localization in Figure 1. In this experiment, we compared wild-type *CDC5* in the *cdc15-2* strain to the *cdc5-1* and *cdc5-2* mutants to characterize the effects of these mutations on late mitosis at the restrictive temperature [20], [21].

When α-factor-synchronized *cdc15-2* cells were released at restrictive temperature (35°C), Bfa1 was detected as a sharp band in G1 phase; the band accumulated as slower-migrating forms during mitosis, and finally attained its maximal phosphorylation states (Figure 1A left). When treated with phosphatase, the slower-migrating forms collapsed into a sharp band (Figure 1A right). The two *cdc5* mutations, *cdc5-1* and *cdc5-2*, affected Bfa1 differently. Bfa1 was phosphorylated as usual in the *cdc5-1* mutant, whereas the *cdc5-2* mutant was severely defective in phosphorylating Bfa1 at the restrictive temperature (Figure 1A; [8]).

We hypothesized that phosphorylation of Bfa1 by Cdc5 might influence its subcellular location. Before examining this possibility we investigated cell cycle progression and the arrest points in *cdc5-1* and *cdc5-2* cells. We shifted G1-synchronized cells to 35°C, and counted every 10 min the number of large budded cells with elongated nuclei stretching along the neck or two segregated nuclei (Figure 1B). Compared to the *cdc15-2* mutant, both *cdc5* mutants exhibited a delay during nuclear elongation (more markedly in *cdc5-2* than in *cdc5-1*), but eventually arrested as large budded cells with separated nuclei, a phenotype similar to that of the *cdc15-2* mutant (Figure 1B). Quantitative analysis of pole-to-pole distances in cells with segregated nuclei revealed that the spindle length relative to cell length was shorter in *cdc5-1* and *cdc5-2* cells than in *cdc15-2* cells; mean spindle length was approximately 83% of that of *cdc15-2* for *cdc5-1*, and 53% for the *cdc5-2* mutant (Figure 1C). These results demonstrate that the *cdc5-1* and *cdc5-2* mutants have defects in nuclear spindle elongation but eventually undergo nuclear division with spindles that are not fully elongated; thereafter they arrest as large budded cells with separate nuclei, as does the *cdc15-2* mutant. Hereafter, for simplicity, we refer to the arrest point of *cdc5-1*, *cdc5-2*, and *cdc15-2* as late anaphase.

We then examined the localization of Bfa1 in *cdc5-1*, *cdc5-2* and *cdc15-2* cells, where Bfa1 and Spc42 were fused to GFP and RFP, respectively. Bfa1-GFP was found on both SPBs immediately after their separation and before the nucleus moved to the bud neck [14], [18]. During metaphase of the *cdc15-2* cells, when the spindle was oriented along the division axis and the nucleus was positioned at the bud neck, Bfa1-GFP was predominantly localized to the SPB closest to the bud neck (Figure 1D; [18]). Bfa1 continued to be selectively localized in *cdc15-2* cells, with elongated dividing nuclei or segregated nuclei (Figure 1D). The *cdc5-1* mutant displayed a Bfa1-GFP localization pattern similar to the *cdc15-2* mutant, whereas Bfa1-GFP remained associated with both SPBs in the *cdc5-2* mutant, even after nuclear segregation (Figure 1D). In anaphase-arrested cells, Bfa1-GFP was present on both SPBs in 90.9±3.0% of *cdc5-2* cells, whereas it was asymmetrically localized on the SPB^d^ in 88.0±1.2% of *cdc15-2* and 92.3±1.5% of *cdc5-1* cells (Figure 1E).

We also observed that the fluorescence intensity of Bfa1-GFP on the SPBs in *cdc5-2* cells was only about 25–28% of that in *cdc15-2* cells (Figure 1F). Recently, Monje-Casas and Amon [18] showed that the intensity of Bfa1-GFP fluorescence is a good measure of the affinity of Bfa1 for the SPB, and its dynamics. We therefore suggest that the phosphorylation of Bfa1 by Cdc5 regulates the dynamics of its behavior, and leads to its asymmetric distribution on the two SPBs.

### A decrease in Bfa1/Bub2 GAP activity is not required for Bfa1 asymmetry

**Figure 2:**
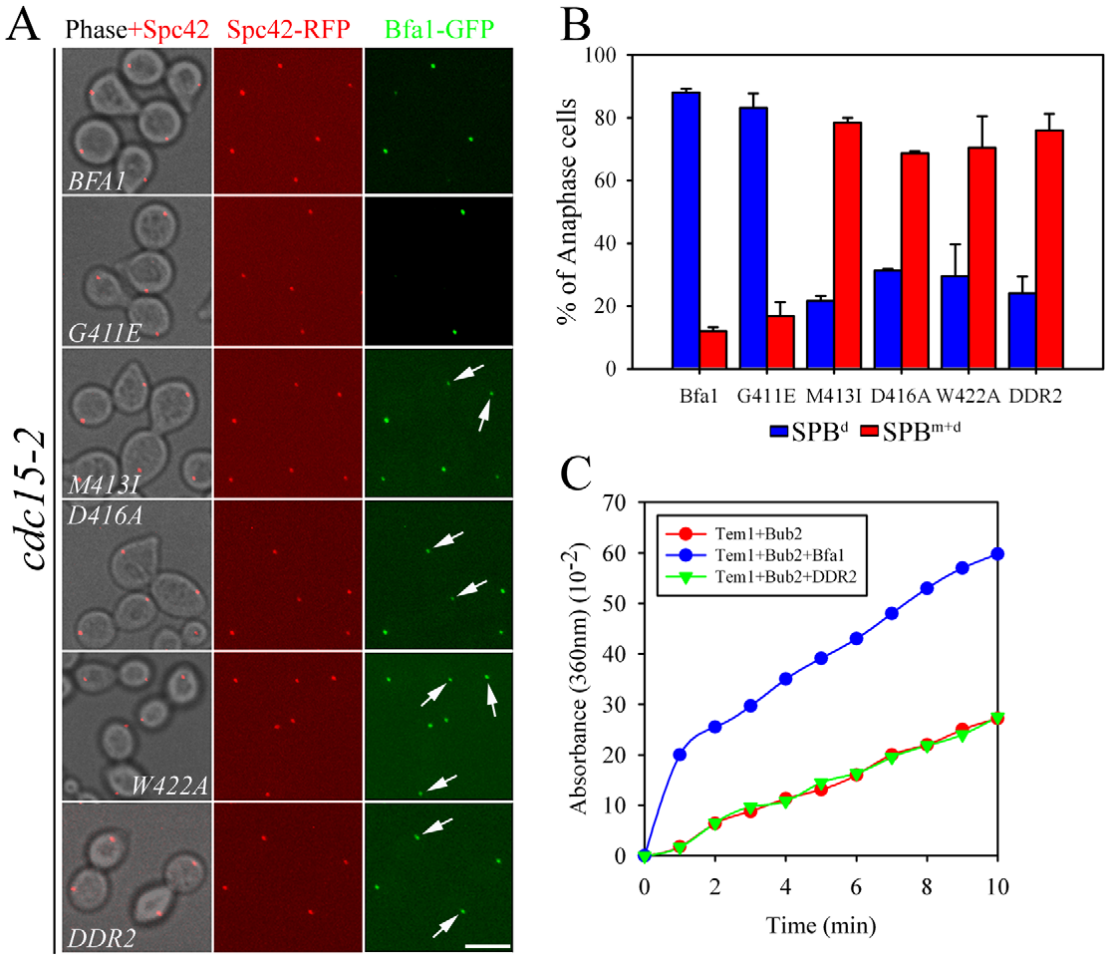
Localization of GAP activity-defective Bfa1 mutants. (A) The anaphase localization of Bfa1 mutants. *cdc15-2SPC42-RFP* cells expressing the indicated *BFA1-GFP* mutants (YSK2545, 2547, 2549, 2551, 2553, and 2557) were synchronized with α-factor at 25°C, released at 35°C for 3 h, and processed for confocal microscopy. (B) The quantification of (A). The average of two independent counts is plotted with standard deviations (n = 200). Arrows indicate Bfa1 localized to the SPB^m^. Bar, 5 µm. (C) *In vitro* GTPase assays of wild-type MBP-Bfa1 and MBP-Bfa1^DDR2^ (5 µg), as described in Materials and Methods.

Since Cdc5 inhibits Bfa1/Bub2 GAP activity toward Tem1 by phosphorylating Bfa1 [9], the presence of Bfa1 on both SPBs in *cdc5-2* cells (Figure 1) could be due to uninhibited Bfa1/Bub2 GAP activity or to the absence of Bfa1 phosphorylation. To distinguish between these possibilities, we examined the localization of GAP activity-defective variants of Bfa1. In *in vitro* Tem1 GTPase assays with Bub2, GAP activity was almost completely absent in Bfa1^W422A^, markedly decreased in both Bfa1^M413I^ and Bfa1^D416A^, and slightly decreased in Bfa1^G411E^ [10]. The *cdc15-2* mutant was used to analyze the localization of each Bfa1 variant at anaphase. We integrated GFP-fused *BFA1*, *BFA1^G411E^*, *BFA1^M413I^*, *BFA1^D416A^*, and *BFA1^W422A^* into *cdc15-2SPC42-RFPΔbfa1* cells, and released these cells from G1 arrest at 35°C. Southern and Western blots verified that the Bfa1 mutants were integrated as single copies and were expressed at similar levels to wild-type Bfa1 (Figure S1). We reasoned that if Bfa1 asymmetry was promoted by inhibition of its GAP activity, the mutant forms of Bfa1 would establish Bfa1 asymmetry prematurely and localize to only one SPB throughout the cell cycle. In fact, however, like wild-type Bfa1, they associated with both SPBs immediately after SPB separation and before nuclear migration to the bud neck (data not shown). In addition, most of the Bfa1^M413I^, Bfa1^D416A^, and Bfa1^W422A^ forms were present on both SPBs even after chromosome segregation, despite their low GAP activities (Figure 2A and 2B). Bfa1^M413I^, Bfa1^D416A^, and Bfa1^W422A^ bound to both SPBs in 78.4±1.7, 68.7±0.6, and 70.5±10.1% of anaphase-arrested cells, respectively, while most wild-type Bfa1 (88.0±1.2%) and Bfa1^G411E^ (83.2±4.5%) was asymmetrically localized to the SPB^d^.

To exclude the possibility that residual GAP activity of Bfa1^M413I^, Bfa1^D416A^, and Bfa1^W422A^ was responsible for their association with both SPBs at anaphase, we constructed another mutant, Bfa1^DDR2^ (Deletion of Direct Repeat 2; Bfa1^G411E M413I D416A W422A^), which was predicted to have no GAP activity; indeed, *in vitro* assays revealed that the Bfa1^DDR2^/Bub2 complex completely failed to stimulate Tem1 GTPase activity (Figure 2C). Consistent with this, Bfa1^DDR2^ was utterly unable to prevent mitotic exit *in vivo* (Figure S2). After confirming single copy integration and normal expression levels (Figure S1), we observed that Bfa1^DDR2^ also persisted at both SPBs in anaphase (76.0±5.3%; Figure 2A and 2B), clearly demonstrating that inhibition of GAP activity does not induce Bfa1 asymmetry. These results suggest that the persistence of Bfa1 on both SPBs in the *cdc5-2* mutant is not due to failure to inhibit GAP activity.

### Bfa1 asymmetry is tightly linked to its phosphorylation by Cdc5

**Figure 3:**
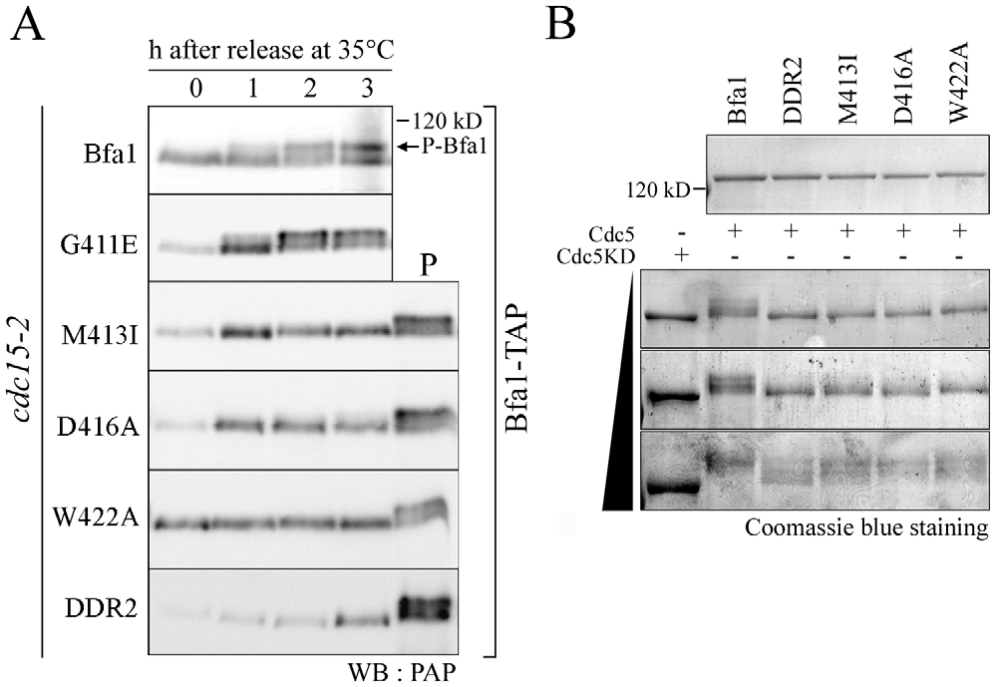
Phosphorylation of GAP activity-defective Bfa1 mutants. (A) Phosphorylation of Bfa1 mutants. *cdc15-2* cells expressing TAP-fused *BFA1* (YSK1153) or mutant *BFA1* (YSK2172, 2173, 2174, 2175, and 2617) were synchronized with α-factor at 25°C, released at 35°C, and harvested at the indicated times. Phosphorylation was detected as reduced electrophoretic mobility. P is a positive control used as in Figure 1A. (B) *In vitro* phosphorylation of Bfa1 mutants by Cdc5. MBP-Bfa1 and MBP-Bfa1 mutants expressed in *E. coli* and purified (shown above) were either untreated (−) or treated (+) with increasing amounts of purified GST-Cdc5 or GST-Cdc5KD.

We then asked if phosphorylation by Cdc5 is required for the asymmetric distribution of Bfa1 on SPBs. We examined phosphorylation of the Bfa1 mutants shown in Figure 2 using SDS-PAGE mobility shift assays in the *cdc15-2* background. When α-factor- synchronized cells were released at 35°C, Bfa1^G411E^ became phosphorylated with similar kinetics to wild-type Bfa1 (Figure 3A). In contrast, slower-migrating forms of Bfa1^M413I^, Bfa1^D416A^, Bfa1^W422A^, and Bfa1^DDR2^ were not detected after the release from G1 arrest (Figure 3A). We did not observe any mobility shift of these Bfa1 mutants even when Cdc5 was overexpressed (Figure S3). Using yeast two-hybrid assays we showed that these Bfa1 mutants interacted with Cdc5 like wild-type Bfa1, demonstrating that the lack of Bfa1 phosphorylation by Cdc5 in these mutants is not due to reduced interaction with Cdc5 (Figure S6B).

To examine the extents of phosphorylation of the Bfa1 mutants, we purified GST-Cdc5 and GST-Cdc5KD (a kinase-dead control) from *S. cerevisiae* and incubated them with MBP-Bfa1 proteins (MBP-Bfa1^M413I^, -Bfa1^D416A^, -Bfa1^W422A^, and -Bfa1^DDR2^) expressed in *E. coli* and purified. As the amount of GST-Cdc5 was increased, wild-type Bfa1 began to appear as multiple, slower migrating forms and eventually appeared as the slowest migrating form, while the Bfa1 mutants remained as multiple, less slowly-migrating forms (Figure 3B). The results of these *in vitro* kinase assays differed slightly from the *in vivo* results in which slower migrating forms of the Bfa1 mutants were rarely seen (Figure 3A; Figure S3). This difference is probably due to either non-specific phosphorylation by the excessive Cdc5 activity used, or the presence of Cdc5 sites that are not easily phosphorylated *in vivo*. In either case, these Bfa1 mutants were obviously resistant to phosphorylation by Cdc5. Note that Bfa1^G411E^ localized asymmetrically to SPB^d^ and was phosphorylated by Cdc5 like wild-type Bfa1, whereas the other Bfa1 variants (Bfa1^M413I^, Bfa1^D416A^, Bfa1^W422A^, and Bfa1^DDR2^) were distributed to both SPBs and were not phosphorylated as efficiently as wild-type Bfa1 by Cdc5. We thus conclude that the asymmetric distribution of Bfa1 is probably linked to its phosphorylation by Cdc5.

### Novel Cdc5 phosphosites are involved in the asymmetric distribution of Bfa1

**Figure 4:**
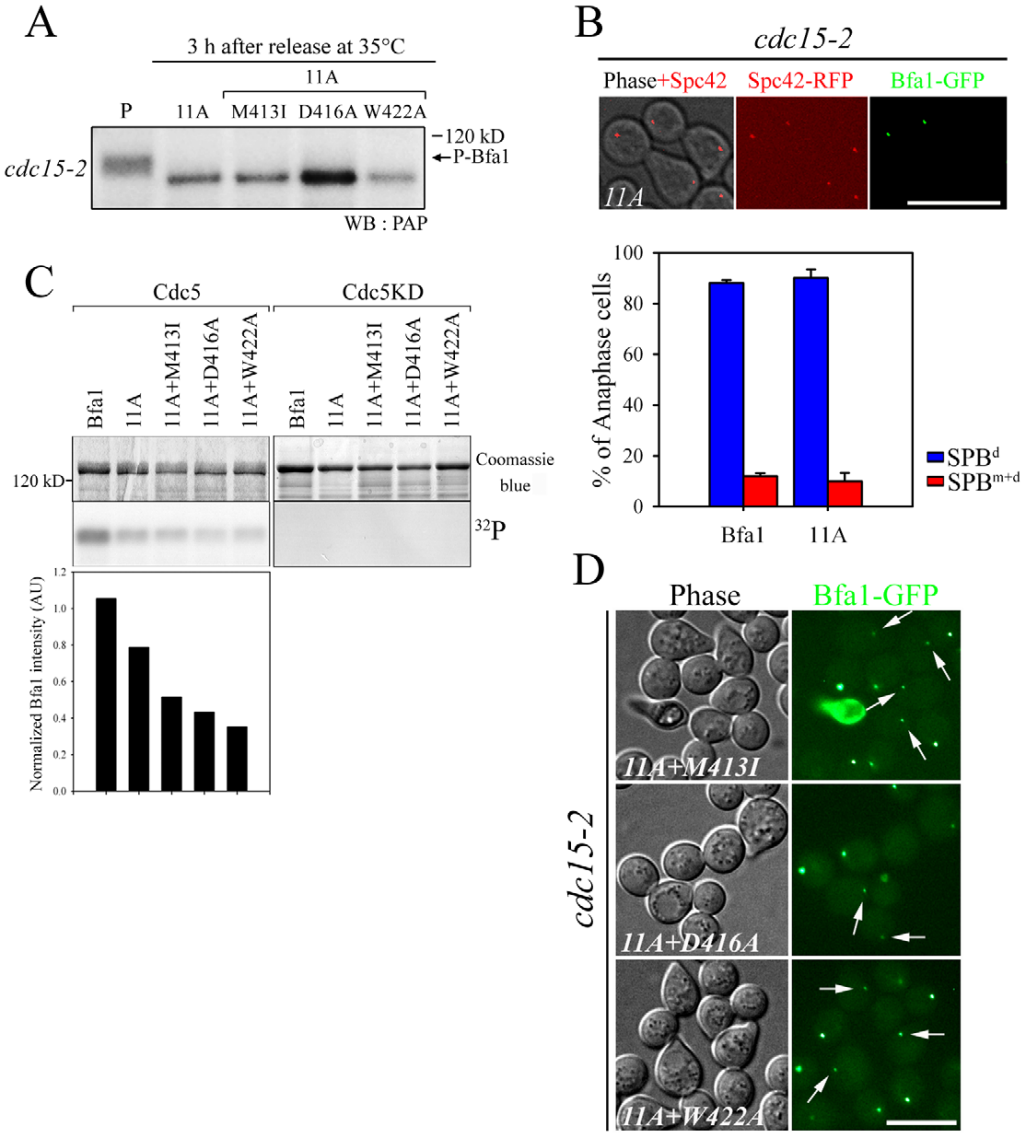
The localization and phosphorylation of Bfa1-11A, Bfa1^M413I^-11A, Bfa1^D416A^-11A, and Bfa1^W422A^-11A. (A) Phosphorylation of Bfa1-11A, Bfa1^M413I^-11A, Bfa1^D416A^-11A, and Bfa1^W422A^-11A at anaphase. The *cdc15-2* cells with indicated *BFA1–TAP* mutants (YSK2177, 2178, 2179, and 2180) were synchronized with α-factor at 25°C, and then released at 35°C for 3 h. P is a positive control used as in Figure 1A. (B and D) Localization of Bfa1-11A, Bfa1^M413I^-11A, Bfa1^D416A^-11A, and Bfa1^W422A^-11A at anaphase. The *cdc15-2* cells with indicated *BFA1-GFP* mutants (YSK2555, 2189, 2190, and 2191) were arrested with α-factor and released at 35°C for 3 h. Bar, 10 µm. (B, graph) Bfa1-11A-GFP association with SPBs was quantified (n = 200). The average of two independent counts is plotted with standard deviation. (C) *In vitro* kinase assay of Bfa1 mutants by Cdc5. MBP-Bfa1 and its mutant derivatives were treated with equivalent amounts of either GST-Cdc5 or GST-Cdc5KD in the presence of γ-[^32^P]-ATP, as described in Materials and Methods. Phosphorylation was detected by autoradiography after SDS-PAGE, and the phosphorylation level was normalized to the intensity of each Bfa1 mutant detected with Coomassie blue. No γ-[^32^P] labeling of substrates was observed when incubated with GST-Cdc5KD. (D) Arrows indicate Bfa1-GFP mutants localized to the SPB^m^.

Eleven Cdc5 phosphosites have been mapped previously, and substituted with Ala in the Bfa1-11A mutant [8]. To confirm the relationship between Bfa1 localization and phosphorylation, we examined the distribution of Bfa1-11A on SPBs. Since Bfa1-11A mobility is not greatly retarded under conditions that normally produce hyperphosphorylated wild-type Bfa1 (Figure 4A), we expected this mutant to be present on both SPBs in anaphase cells. However, most of the Bfa1-11A (90.1±3.4%) was only associated with SPB^d^, like wild-type Bfa1 (88.0±1.25%) (Figure 4B). We therefore examined whether Bfa1-11A was further phosphorylated by Cdc5. When MBP-fused Bfa1-11A was incubated with GST-Cdc5 and γ-[^32^P] ATP, ^32^P incorporation was observed, whereas no ^32^P incorporation was detected in reactions with an equivalent amount of GST-Cdc5KD (Figure 4C). These observations are consistent with a previous report that mutation of these 11 residues reduces *in vitro* phosphorylation of Bfa1 by only 75% [8], and demonstrate that not all Cdc5 phosphorylation sites are mutated in Bfa1-11A.

Based on these results, we reasoned that there are unidentified Cdc5 phosphosites and that these could be responsible for the asymmetric distribution of Bfa1-11A. We further hypothesized that these novel sites are not efficiently phosphorylated on Bfa1^M413I^, Bfa1^D416A^, and Bfa1^W422A^, thereby causing these Bfa1 variants to persist at both SPBs in anaphase. If that were the case, the introduction of the M413I, D416A, or W422A mutation into Bfa1-11A should impair Bfa1-11A asymmetry and reduce its phosphorylation. Indeed, ^32^P incorporation into Bfa1^M413I^-11A, Bfa1^D416A^-11A, and Bfa1^W422A^-11A was less efficient than into Bfa1-11A (Figure 4C), and Bfa1^M413I^-11A, Bfa1^D416A^-11A, and Bfa1^W422A^-11A were each symmetrically distributed to both SPBs (Figure 4D). Consistent with this, when Cdc5 was overexpressed to force phosphorylation of Bfa1 by Cdc5, we detected a mobility shift in Bfa1-11A but not in Bfa1^M413I^-11A, Bfa1^D416A^-11A, or Bfa1^W422A^-11A (Figure S4A). However, in the anaphase-arrested *cdc15-2* background, the mobilities of Bfa1^M413I^-11A, Bfa1^D416A^-11A, and Bfa1^W422A^-11A were nearly the same as that of Bfa1-11A (Figure 4A), suggesting that phosphorylation of the residue(s) responsible for asymmetry is not efficient in cells expressing endogenous Cdc5 levels. Together, these results support the presence of unidentified Cdc5 target residues required for establishing Bfa1 asymmetry.

### Mutation of ^452^S, ^453^S, ^454^S, and ^559^S disrupts Bfa1 asymmetry

**Figure 5:**
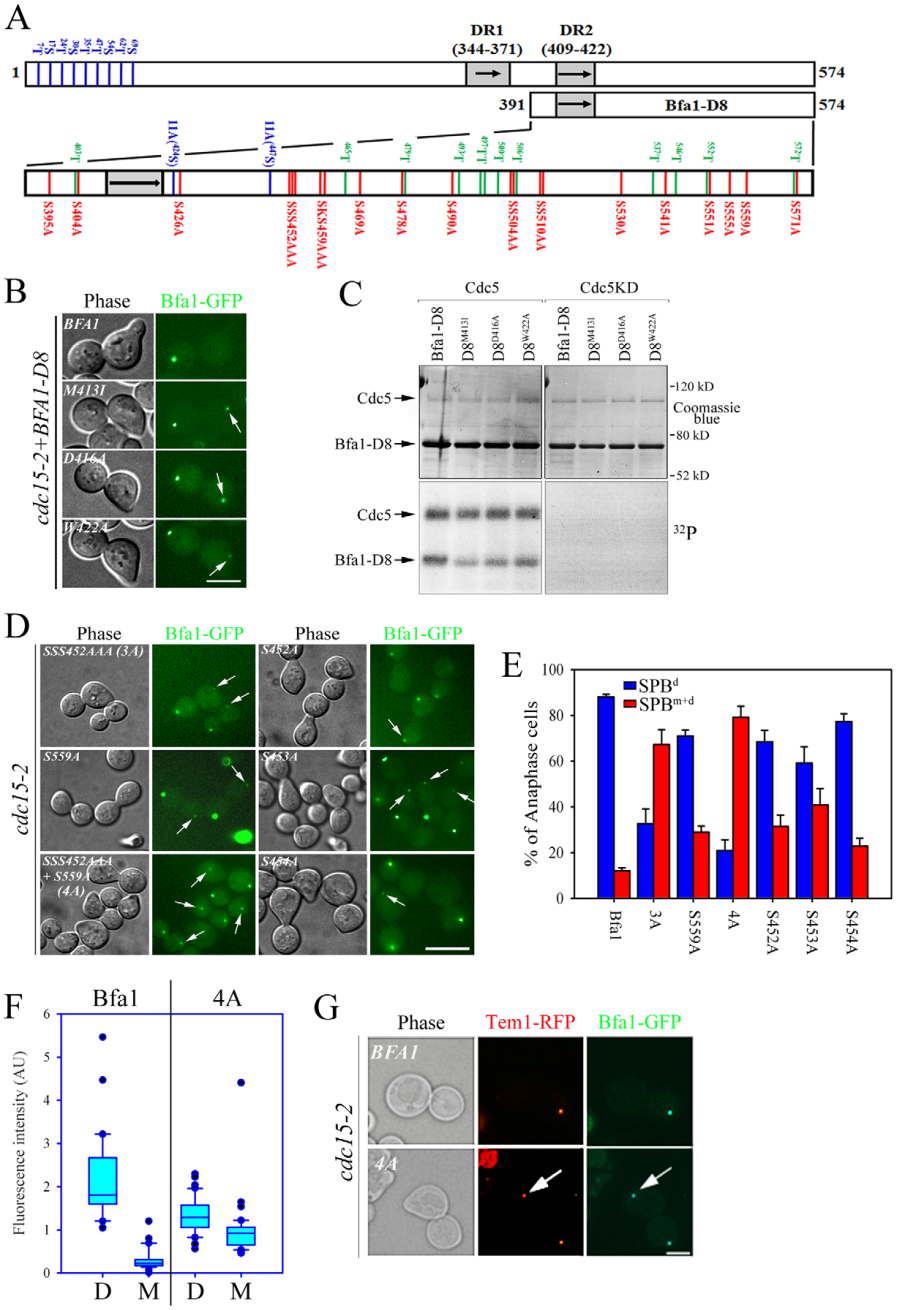
Identification of putative phosphosite(s) regulating the asymmetric localization of Bfa1. (A) Schematic diagram of full-length Bfa1 and Bfa1-D8. The position of Ser and Thr residues in Bfa1-D8 is shown. The arrows and shaded boxes represent two regions of imperfect direct repeats (DR1 and DR2) that are highly conserved among fungi (Kim *et al.*, 2008). The Ser and Thr residues that are mutated to Ala in Bfa1-11A are shown in blue. Ser residues that were mutated to Ala in this study are shown in red, and Thr are shown in green. (B,D) The localization of Bfa1-D8 mutants (B) and Bfa1 Ser mutants (D) at anaphase. α-factor synchronized cells were released at 35°C for 3 h, and observed by fluorescence microscopy. Arrows indicate Bfa1 localized to the SPB^m^. Bar, 5 µm. (B) YSK2195, 2196, 2197, 2198 strains and (D) YSK2192, 2194, 2330, 2332, 2193 and 2334 were used. (C) *In vitro* kinase assay of Bfa1-D8 mutants by Cdc5. The experiment was performed as described in Figure 4C. No γ-[^32^P] labeling of substrates was observed from reactions with GST-Cdc5KD. (E) The quantification of (D). Anaphase cells with the indicated phenotype were counted (n = 200). The average of two independent counts is plotted with standard deviation. (F) Box plots comparing the fluorescence intensities of Bfa1-GFP at SPBs. *cdc15-2SPC42-RFPBFA1-GFP* (YSK2545) and *cdc15-2SPC42-RFP BFA1^4A^-GFP* (YSK2561) cells were released from α-factor arrest for 3 h at 35°C. GFP fluorescence signals were analyzed as described in Materials and Methods (n = 30 for Bfa1 and 40 for Bfa1^4A^). The line inside the box indicates the median. D, GFP signal at the SPB^d^. M, GFP signal at the SPB^m^. (G) The anaphase localization of Tem1-RFP in *BFA1* and *BFA1^4A^* cells. *cdc15-2TEM1-RFPBFA1-GFP* (YSK2720) and *cdc15-2TEM1-RFP Bfa1^4A^-GFP* (YSK2721) cells were synchronized with α-factor at 25°C, released at 35°C for 3 h, and observed by fluorescence microscopy. Arrows indicate Bfa1-GFP and Tem1-RFP localized to the SPB^m^. Bar, 5 µm.

We previously showed that the C-terminal 184 residues of Bfa1 (Bfa1-D8^391–574^) sufficiently inhibit the MEN and localize predominantly to the SPB^d^ (Figure 5A and 5B; [22]). As expected, GFP-fused Bfa1-D8^M413I^, Bfa1-D8^D416A^, and Bfa1-D8^W422A^ were found at both SPBs (Figure 5B). In addition, the Bfa1-D8 mutants were less efficiently phosphorylated by Cdc5; Bfa1-D8 was more intensely labeled by ^32^P and detected in a slower-migrating form than Bfa1-D8^M413I^, Bfa1-D8^D416A^, and Bfa1-D8^W422A^ (Figure 5C). This indicated that the putative Cdc5 target site(s) responsible for Bfa1 asymmetry was probably located within the C-terminal 184 residues of Bfa1.

We next searched for possible kinase targets within the C-terminal 184 residues of Bfa1. Cdc5 is a Ser/Thr protein kinase, and Bfa1-D8 contains 23 Ser and 12 Thr residues (Figure 5A). We first systematically mutated 21 of the 23 Ser residues to Ala in Bfa1-D8 and constructed 16 different Ser mutants as GFP fusion proteins: Bfa1^S395A^, Bfa1^S404A^, Bfa1^S426A^, Bfa1^SSS452AAA^, Bfa1^SKS459AAA^, Bfa1^S469A^, Bfa1^S478A^, Bfa1^S490A^, Bfa1^SS504AA^, Bfa1^SS510AA^, Bfa1^S530A^, Bfa1^S541A^, Bfa1^S551A^, Bfa1^S555A^, Bfa1^S559A^, and Bfa1^S571A^ (Figure 5A, Table 1). Both ^424^S and ^447^S were excluded because they were included in Bfa1-11A. We integrated each of the Ser mutants into *cdc15-2Δbfa1* cells, and examined their localization at anaphase. The results are summarized in Table 1. Most of the GFP-fused Ser mutants exhibited the localization pattern of wild-type Bfa1-GFP (Table 1; and data not shown). However, the percentage of anaphase-arrested cells with Bfa1-GFP on both SPBs was significantly increased in *cdc15-2BFA1^SSS452AAA^* (for simplicity, *cdc15-2BFA1^3A^*) and *cdc15-2BFA1^S559A^* cells; Bfa1-GFP was present on both SPBs in 12.0±1.2% of *cdc15-2BFA1* cells, 67.3±6.5% of *cdc15-2BFA1^3A^* cells, and 29±2.6% of *cdc15-2BFA1^S559A^* cells (Figure 5D and 5E, Table 1). Cells expressing the substitutions of each Ser in Bfa1^3A^, GFP-tagged Bfa1^S452A^, Bfa1^S453A^, or Bfa1^S454A^, also had larger numbers of anaphase cells with Bfa1-GFP on both SPBs (Table 1), demonstrating that all three Ser residues contribute to Bfa1 asymmetry. In contrast, mutation of some of the Thr residues to Ala around the newly identified ^452^S, ^453^S, ^454^S, and ^559^S sites (^465^T, ^497^TT, ^500^T, ^537^T, ^546^T, ^552^T, and ^572^T; Figure 5A) had little effect on Bfa1 asymmetry (Table 1; data not shown).

**Table 1:** Summary of Bfa1 localization on SPBs in various Bfa1 mutants.

Strains	Mutation sites	Localization to both SPBs	Strains	Mutation sites	Localization to both SPBs
Bfa1	Wild type	12±1.2%	Bfa1^S395A^	S395A	As wild type
Bfa1 D8			Bfa1^S404A^	S404A	As wild type
Bfa1^G411E^	G411E	16.8±4.5%	Bfa1^S426A^	S426A	As wild type
Bfa1^M413I^	M413I	78.4±1.7%	Bfa1^SKS459AAA^	S459A K460A S461A	As wild type
Bfa1 D8^M413I^			Bfa1^S469A^	S469A	As wild type
Bfa1^D416A^	D416A	68.7±0.6%	Bfa1^S478A^	S478A	As wild type
Bfa1 D8^D416A^			Bfa1^S490A^	S490A	As wild type
Bfa1^W422A^	W422A	70.5±10.1%	Bfa1^SS504AA^	S504A S505A	As wild type
Bfa1 D8^W422A^			Bfa1^SS510AA^	S510A S511A	As wild type
Bfa1^DDR2^	G411E M413I D416A W422A	76±5.3%	Bfa1^S530A^	S530A	As wild type
Bfa1^11A M413I^	11A+M413I	As Bfa1^M413I^	Bfa1^S551A^	S551A	As wild type
Bfa1^11A D416A^	11A+D416A	As Bfa1^D416A^	Bfa1^S555A^	S555A	As wild type
Bfa1^11A W422A^	11A+W422A	As Bfa1^W422A^	Bfa1^S571A^	S571A	As wild type
Bfa1^3A^	S452A S453A S454A	67.3±6.5%	Bfa1^T465A^	T465A	As wild type
Bfa1^S452A^	S452A	31.5±4.9%	Bfa1^T497A^	T497A	As wild type
Bfa1^S453A^	S453A	40.9±7.1%	Bfa1^T500A^	T500A	As wild type
Bfa1^S454A^	S454A	22.8±3.5%	Bfa1^T537A^	T537A	As wild type
Bfa1^S559A^	S559A	29±2.6%	Bfa1^T546A^	T546A	As wild type
Bfa1^4A^	S452A S453A S454A S559A	79.2±4.8%	Bfa1^T552A^	T552A	As wild type
Bfa1^4D^	S452D S453D S454D S559D	0.7±1.5%	Bfa1^T572A^	T572A	As wild type
Bfa1^11A^	T7A S17A T24A S30A T35A T47A S54A T62A S69A S424A S447A	9.9±3.4%			

To confirm these results, we constructed Bfa1^4A^, in which all four Ser residues were substituted with Ala (SSS452AAA S559A referred to as 4A). Bfa1^4A^-GFP was detected on both SPBs in 79.2±4.8% of anaphase cells, compared with 67.3±6.5% for *cdc15-2BFA1^3A^* and 29±2.6% for *cdc15-2BFA1^S559A^* cells, again showing that all four Ser residues play a role in establishing Bfa1 asymmetry (Figure 5D and 5E). Quantification of the GFP signal in anaphase-arrested cells showed that the fluorescence intensity of Bfa1^4A^-GFP was nearly the same at both SPBs, weaker than that of wild-type Bfa1-GFP at the SPB^d^ (Figure 5F) and similar to that of Bfa1-GFP in the *cdc5-2* mutant (Figure 1).

In order to verify that the symmetric localization of Bfa1^4A^ was not caused by defective interaction with Cdc5, we compared the physical interactions of Bfa1^4A^ and wild-type Bfa1 with Cdc5 using yeast two-hybrid assays. As shown in Figure S6A, Bfa1^4A^ interacted as strongly with Cdc5 as wild-type Bfa1. The asymmetric localization of Tem1 to the daughter SPB in anaphase depends on Bfa1 and Bub2 [14]. Therefore we asked whether the presence of the Bfa1^4A^ mutant on both SPBs affected the asymmetric localization of Tem1. To answer this question we integrated GFP-fused *BFA1^4A^* or *BFA1* into *cdc15-2TEM1-RFPΔbfa1* cells, arrested these cells in G1, and released them at 35°C. As expected, Tem1-RFP followed the localization pattern of Bfa1-GFP in anaphase: it was present on both SPBs in the Bfa1^4A^ background and distributed asymmetrically to the SPB^d^ in the wild-type background (Figure 5G).

### ^452^S, ^453^S, ^454^S, and ^559^S can be phosphorylated by Cdc5 *in vivo* and *in vitro*

**Figure 6:**
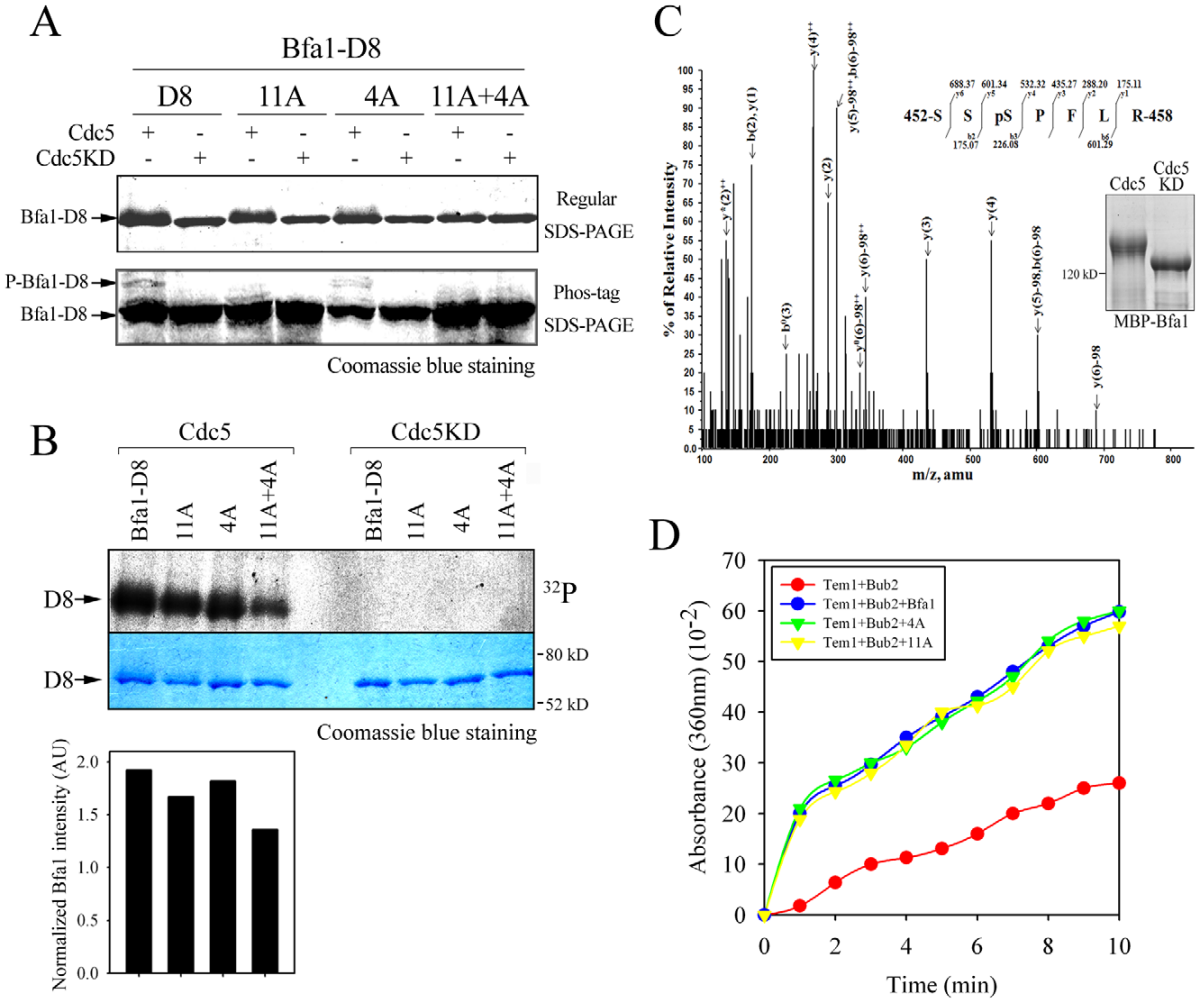
The phosphorylation and GAP activity of Bfa1^4A^ mutant. (A) *In vitro* phosphorylation of Bfa1-D8 mutants by Cdc5. MBP-Bfa1-D8 and MBP-Bfa1-D8 mutants were either untreated (−) or treated (+) with equivalent amounts of either GST-Cdc5 or GST-Cdc5KD, and stained by Coomassie blue after regular SDS-PAGE (top) and Phos-tag SDS-PAGE (bottom). Phosphorylation was detected as reduced electrophoretic mobility (top) and phospho-Bfa1-D8 bands (bottom). (B) *In vitro* kinase assay of Bfa1-D8 mutants by Cdc5. Bfa1-D8 and its mutant derivatives were treated with equivalent amounts of either GST-Cdc5 or GST-Cdc5KD in the presence of γ-[^32^P]-ATP, as described in Materials and Methods. Phosphorylation of D8 and its mutants was detected by autoradiography after SDS-PAGE (top), and the phosphorylation level was normalized to the intensity of each Bfa1 mutant detected with Coomassie blue staining (bottom). No γ-[^32^P] labeling of substrates was observed from reactions with GST-Cdc5KD. (C) Determination of Cdc5-dependent phosphorylation by mass spectrometry. In-gel tryptic digests of the *in vitro* phosphorylated Bfa1 by purified Cdc5 kinase were analyzed by LC-MS/MS. The MS/MS spectra of doubly charged mass/charge (m/z) = 437.186^2+^ were used to search against a limited database containing only the protein of interest, Bfa1, and corresponds to a Bfa1 peptide ^452^SSpSPFLR^458^ with a phosphorylated Ser^454^ residue. The monoisotopic mass of the neutral peptide is 872.357. The b and y ions detected are marked on the peaks. The doubly charged ions were marked as ++ on the peaks. The mass of 98 on the peaks was derived from neutral losses (−97.9769 Da) of phosphoric acid from the precursor ion. Peaks are seen for ions which have lost ammonia (−17 Da), denoted by y*, and water (−18 Da), denoted by y° and b°. (D) *In vitro* Tem1 GTPase assay. This experiment was performed in parallel with Figure 2C, and thus, the wild-type Bfa1/Bub2 and Bub2 controls are the same in both cases. 5 µg MBP-Bfa1, MBP-Bfa1^4A^, or MBP-Bfa1-11A were included in each reaction mixture.

We then asked if ^452^S, ^453^S, ^454^S, and ^559^S of Bfa1 are phosphorylated by Cdc5. Measuring phosphorylation of these four Ser without phosphorylation of the 11 known targets was not feasible. Therefore, since the N-terminus of Bfa1 contains nine of the Cdc5 targets of Bfa1-11A, we used the C-terminal 184 residues of Bfa1 in *in vitro* kinase assays. MBP-tagged Bfa1-D8, Bfa1-D8-11A, Bfa1-D8^4A^, and Bfa1-D8^4A^-11A were incubated with GST-Cdc5 or GST-Cdc5KD. The extents of phosphorylation were determined from the resulting mobility shifts and phospho-Bfa1-D8 bands by Phos-tag SDS-PAGE (Figure 6A). The band representing Bfa1-D8^4A^-11A was tighter in mobility shifts and the phospho-Bfa1-D8^4A^-11A forms were not detected in Phos-tag SDS-PAGE (Figure 6A). The extents of phosphorylation were further confirmed by measuring γ-[^32^P] incorporation and Bfa1-D8^4A^-11A less intensely labeled with ^32^P than that of Bfa1-D8-11A (Figure 6B), demonstrating that the 4A mutations reduce Bfa1 phosphorylation by Cdc5. In addition, we speculated that the slower-migrating forms of Bfa1-11A observed in *CDC5*-overexpressing cells (Figure S4A) resulted, at least in part, from the phosphorylation of residues, such as ^452^S, ^453^S, ^454^S, and S^559^, required for Bfa1 asymmetry. Indeed, the 4A mutations abolished the slower migrating forms of Bfa1-11A in cells overexpressing *CDC5* (Figure S4A), consistent with the results in Figure 6A and 6B. Thus, we suggest that Cdc5 phosphorylates ^452^S, ^453^S, ^454^S, and ^559^S of Bfa1.

To confirm this we used mass spectrometry (MS) to map Bfa1 phosphorylation sites (Figure 6C). Recombinant MBP-Bfa1 was phosphorylated *in vitro* with GST-Cdc5 or with GST-Cdc5KD as a negative control (Figure 6C). Subsequently, MBP-Bfa1 was purified by SDS-PAGE, excised, digested with trypsin, and analyzed by phosphopeptide-selective precursor ion-scanning liquid chromatography (LC) MS. The success of this approach was assessed by seeing if we could detect the 11 previously identified Cdc5 phosphorylation sites. Although we were unable to identify eight of the 11 phosphorylation residues of Bfa1, likely due to their presence on extremely small (^547^S-^549^K; three amino acids) or large (^28^F-^75^K; 48 amino acids) trypsin peptides, we did detect phosphorylated forms of ^17^S, ^24^T, and ^447^S with high frequency. The same tandem LC-MS/MS analysis identified two phosphopeptide species, ^452^SSpSPFLR^458^ (pS, phosphorylated S; Figure 6C) and ^452^SpSpSPFLR^458^ (data not shown), containing ^453^S and ^454^S, two of the four Bfa1 phosphorylation residues responsible for asymmetry identified above. Phosphorylation of ^452^S and ^559^S was not detected by this approach.

To ask if the GAP activity of Bfa1^4A^ affects its association with both SPBs at anaphase, we directly measured the GAP activity of Bfa1^4A^/Bub2. When MBP-Bfa1^4A^ was added to the reaction together with Tem1 and GST-Bub2, as in the experiment of Figure 2C, γ-P_i_ increased rapidly with kinetics similar to those obtained with wild-type Bfa1, indicating that the 4A substitutions had no effect on Bfa1 GAP activity (Figure 6D). We also examined the control of mitotic exit by Bfa1^4A^ *in vivo*: *BFA1^4A^* cells arrested as large budded cells, as did wild-type *BFA1* cells, in the presence of various checkpoint-activating signals (Figure S5A–S5C). Together, these results show that the Bfa1^4A^ has functional GAP activity and demonstrate that the presence of Bfa1^4A^ at both SPBs is independent of GAP activity.

### Asymmetric localization of Bfa1 fine-tunes the timing of MEN activation

**Figure 7:**
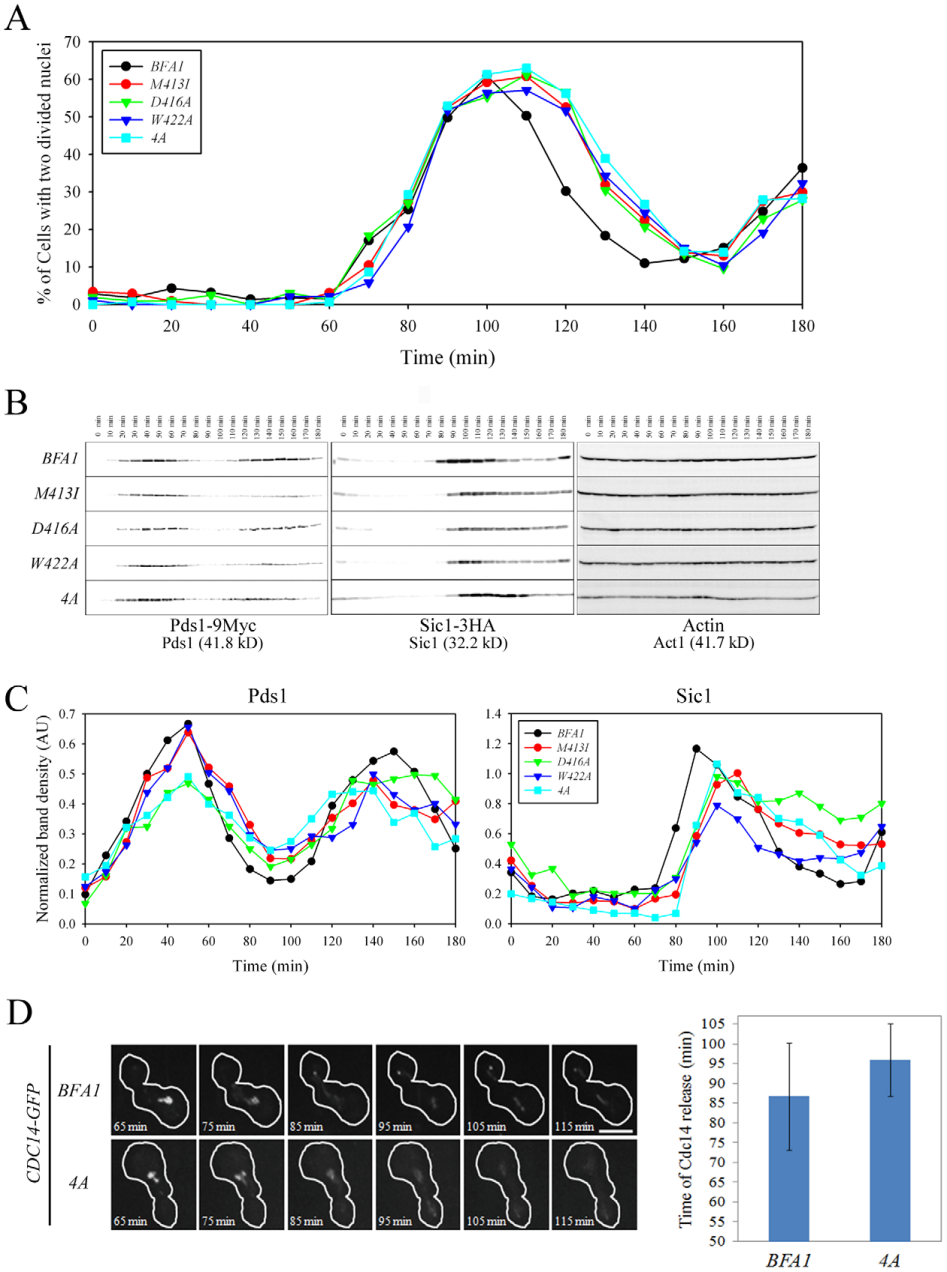
Cell cycle progression in mutant cells with symmetric localization of Bfa1. (A–C) The indicated cells (YSK2202, 2204, 2205, 2206 and 2338) were grown to mid-log phase, synchronized in G1 with α-factor, and then released into fresh medium at 25°C. (A) Cell cycle progression. Samples were collected every 10 min. Cells were stained with DAPI and large-budded cells with divided nuclei were counted (n = 200). (B and C) Pds1 and Sic1 levels. (B) Samples were collected every 10 min. To analyze changes in Pds1 and Sic1 levels during cell cycle progression, protein extracts were prepared and subjected to western blotting with anti-HA and anti-Myc. Actin served as a loading control. (C) Calibration curves were used to quantify Pds1 and Sic1 levels in (B). The band intensity of Pds1 and Sic1 was normalized to Actin. (D) Cdc14 release in live wild-type *BFA1* and asymmetry-defective *BFA1^4A^* mutant cells. The indicated cells (YSK2854 and YSK2855) were grown to mid-log phase, synchronized in G1 with α-factor, released into fresh medium at 25°C, and monitored using time-lapse confocal microscopy. Snap shots of every 10 min were shown after released for 65 min. The average time of Cdc14 release was plotted in *BFA1* and *BFA1^4A^* mutant cells (n = 9 for *BFA1* and 6 for *BFA1^4A^*). Bar, 5 µm.

Since Bfa1 is found on both SPBs in cells with misaligned spindles [16], it has been proposed that symmetrically localized Bfa1, and in particular, the Bfa1 associated with the SPB in mother cells, contributes to the arrest of mitotic exit [17]. However, the *BFA1^M413I^*, *BFA1^D416A^*, *BFA1^W422A^*, and *BFA1^4A^* cells grew well and did not show any apparent cell cycle delay in unperturbed conditions, despite having Bfa1 on both SPBs throughout the cell cycle. To better understand the function of Bfa1 asymmetry in mitotic exit, we examined cell cycle progression in these asymmetry-defective *BFA1* mutants. Following G1 synchronization and release at room temperature, we monitored large budded cells with two divided nuclei (for simplicity, anaphase cells). In both wild-type and mutant *BFA1* cultures, anaphase cells began to accumulate approximately 70 min after release (Figure 7A). In *BFA1* cells, numbers of anaphase cells began to decrease about 110 min after release. Interestingly, in the *BFA1^M413I^*, *BFA1^D416A^*, *BFA1^W422A^*, and *BFA1^4A^* cultures, the decrease in anaphase cells was delayed by about 10 min (Figure 7A).

To further examine the delay in cell cycle progression in the Bfa1 mutant cells, we measured Pds1 and Sic1 levels. Pds1 is an anaphase inhibitor that is degraded upon sister chromatid separation [23] and Sic1 is a negative regulator of mitotic CDKs that accumulates following activation of the MEN (For a review, see [24]). Consistent with Figure 7A, the wild-type and all the *BFA1* mutants exhibited a drop in Pds1 levels approximately 60 min after release (Figure 7B and 7C), indicating that Bfa1 asymmetry and its persistent association with the SPB^m^ does not alter the timing of anaphase onset. Importantly, Sic1 accumulation in the wild-type began about 80 min after release, whereas it began about 90 min after release in *BFA1^M413I^*, *BFA1^D416A^*, *BFA1^W422A^*, and *BFA1^4A^* cells (Figure 7B and 7C). *BFA1^G411E^* cells, with a normal Bfa1 distribution, displayed the Pds1 and Sic1 kinetics of the wild-type (data not shown). These results show that asymmetry-defective *BFA1* cells activate the MEN approximately 10 min later than cells with normal Bfa1 localization.

We confirmed the 10 min delay of mitotic exit in *BFA1^4A^* mutant cells by examining the dynamics of Cdc14 release in *BFA1* and *BFA1^4A^* mutant cells by live cell analysis using time-lapse confocal microscopy. Mitotic exit requires full activation of Cdc14 by releasing it from the nucleolus in late anaphase [25]. As shown in the captured images of Figure 7D, Cdc14 was fully released out of the nucleolus 95 and 105 min after *BFA1^4A^* mutant cells were released from G1 arrest, while wild-type *BFA1* cells released Cdc14 approximately 10 min earlier. When we examined time lapse images of several *BFA1^4A^* and wild-type cells, the average time of Cdc14 release was 95.8±9.1 min after release from G1 arrest in the *BFA1^4A^* mutant and 86.7±13.5 min in *BFA1* wild-type (Figure 7D). These observations clearly demonstrate that in the asymmetry-defective *BFA1^4A^* cells the activation of MEN is delayed by approximately 10 min compared with wild-type *BFA1* cells.

We then investigated the physical interaction of Bfa1^4A^ with Tem1 and Bub2 using yeast two-hybrid assays to verify that the 10 min delay of mitotic exit was due to its symmetric localization resulting from lack of phosphorylation and not to any reduction in its interaction with Tem1 and Bub2. As shown in Figure S6C and S6D, Bfa1^4A^ interacted with Tem1 and Bub2 like wild-type Bfa1. Together, these observations demonstrate that Cdc5-dependent phosphorylation of the four identified serine residues in Bfa1 controls its displacement from the SPB^m^ for timely mitotic exit in unperturbed mitosis.

### Phospho-mimicking of ^452^S, ^453^S, ^454^S, and ^559^S results in Bfa1 asymmetry and timely mitotic exit

**Figure 8:**
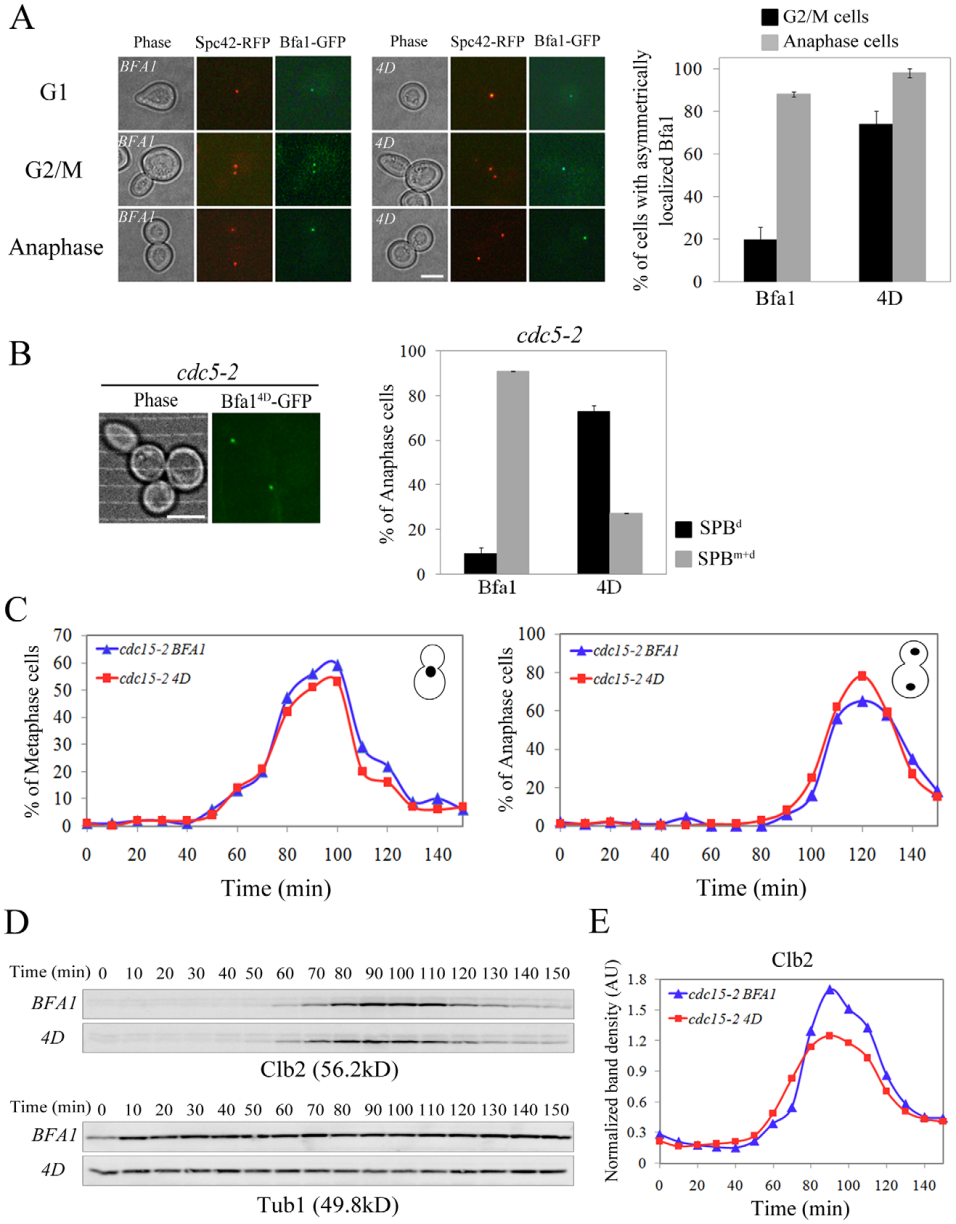
Localization and cell cycle progression of a phospho-mimetic Bfa1^4D^ mutant. (A,B) Localization of phospho-mimetic Bfa1^4D^ in *cdc15-2* and *cdc5-2* mutant cells. *cdc15-2SPC42-RFPBFA1-GFP* (YSK2545), *cdc15-2SPC42-RFPBFA1^4D^-GFP* (YSK2858) and *cdc5-2BFA1^4D^-GFP* (YSK 2907) cells were released from α-factor arrest at 35°C. (A) The localization of Bfa1-GFP were examined using fluorescence microscopy at G1 (0 min), G2/M (60 min), and anaphase (180 min) after the release. Among *BFA1-GFP* (YSK2545) and *BFA1^4D^-GFP* (YSK2858) cells in G2/M (n = 157 for *BFA1* and 132 for *BFA1^4D^*) and late anaphase (n = 200 for *BFA1* and 700 for *BFA1^4D^*), cells with asymmetrically localized Bfa1-GFP were counted. The average percentage of cells with asymmetrically localized Bfa1-GFP in two independent counts is plotted with standard deviation. Bar, 5 µm. (B) The localization of Bfa1^4D^ in *cdc5-2* mutant cells was observed by confocal microcopy. The average percentage of cells with asymmetrically localized Bfa1-GFP in two independent counts is plotted with standard deviation (n = 128 for *BFA1^4D^*). Bar, 5 µm. (C–E) Cell cycle progression of phospho-mimetic *BFA1^4D^*. *BFA1-GFP* (YSK2545) and *BFA1^4D^-GFP* (YSK2858) cells were grown to mid-log phase, synchronized in G1 with α-factor, and released into fresh medium at 25°C. Samples were collected every 10 min. (C) Cells were stained with DAPI and the percentage of indicated cell types was determined (n = 100). (D,E) Clb2 levels during the cell cycle. (D) The samples of (C) were subjected to western blotting with anti-Clb2. Tub1 (α-tubulin) served as a loading control. (E) The Clb2 band intensity in (D) was normalized to Tub1 and plotted to quantify Clb2 levels in (D).

In order to confirm that Cdc5-dependent phosphorylation of ^452^S, ^453^S, ^454^S, and ^559^S in Bfa1 controls the asymmetric localization of Bfa1 for timely mitotic exit, we constructed Bfa1^4D^ (SSS452DDD S559D referred to as 4D) that mimics the negative charges due to phosphorylation. GFP-fused *BFA1^4D^* was integrated into *cdc15-2SPC42-RFPΔbfa1* cells, and as shown in Figure 8A, Bfa1^4D^-GFP remained localized asymmetrically on the SPBs throughout the cell cycle, even in G2/M when wild-type Bfa1 is present on both SPBs. At G2/M, the cells with asymmetrically localized Bfa1 on the SPBs were significantly increased in *cdc15-2BFA1^4D^* cells than in *cdc15-2BFA1* cells; 73.9±6.3% of *cdc15-2BFA1^4D^* and 19.7±5.8% of *cdc15-2BFA1* cells (Figure 8A). When cells were arrested in late anaphase, Bfa1^4D^-GFP was asymmetrically localized on the daughter SPB in 98.6±1.5% of the cells, compared to 20.8±4.8% in *cdc15-2BFA1^4A^* cells (Figure 8A and Figure 5E). These observations demonstrate that symmetric localization of Bfa1^4A^ is caused by loss of phosphorylation, and suggest that phosphorylation of Bfa1 by Cdc5 on ^452^S, ^453^S, ^454^S, and ^559^S is necessary for establishing Bfa1 asymmetry.

In kinase-defective *cdc5-2* cells, Bfa1 was unphosphorylated and localized to both SPBs (Figure 1D). The finding that Cdc5-dependent Bfa1 phosphorylation on ^452^S, ^453^S, ^454^S, and ^559^S residues regulates its asymmetric localization prompted us to ask whether the phospho-mimicking Bfa1^4D^ is asymmetrically located in *cdc5-2* cells. For this, we integrated *pRS304-BFA1^4D^-GFP* into the TRP1 locus of *cdc5-2Δbfa1* cells. In late anaphase-arrested *cdc5-2* cells, Bfa1^4D^ was asymmetrically localized in 72.7±0.1% of the cells, while Bfa1-GFP was present on both SPBs in 90.9±3.0% of cells (Figure 1E and Figure 8B). These observations further support the notion that Cdc5-dependent phosphorylation of ^452^S, ^453^S, ^454^S, and ^559^S in Bfa1 is important for its asymmetric localization.

Since Bfa1 is a target of Cdc5 phosphorylation for triggering mitotic exit, its deletion has been reported to rescue kinase-defective *cdc5-2* cells arrested in late anaphase at restrictive temperatures [8]. To see whether Bfa1^4D^ can inhibit the MEN, we tested whether the viability of *cdc5-2* could be restored by *BFA1^4D^*. As shown in Figure S7, *BFA1^4D^* as well as wild-type *BFA1* suppressed the growth of *cdc5-2Δbfa1* cells, while knock-out of *BFA1* rescued the viability of *cdc5-2* cells. These results demonstrate that Bfa1^4D^ is able to inhibit MEN like wild-type Bfa1.

Since Bfa1^4D^-GFP is exclusively localized on one of the SPBs during mitosis (Figure 8A) but functions as a negative regulator of the MEN, we asked whether the lack of dynamic localization of phospho-mimetic Bfa1^4D^ affects cell cycle progression. *cdc15-2* cells expressing wild-type *BFA1* or phospho-mimetic *BFA1^4D^* were synchronized in G1 and released at room temperature, and their cell cycle progression was monitored by counting metaphase and anaphase cells. As shown in Figure 8C, *BFA1^4D^* cells exhibited the same kinetics of cell cycle progression as wild-type *BFA1* cells. In both wild-type and *BFA1^4D^* cells, metaphase cells began to accumulate at 60 min and peaked at 100 min, while anaphase cells appeared at 100 min and reached a peak at 120 min after release (Figure 8C).

To further analyze the cell cycle progression, we measured the mitotic cyclin Clb2, which is degraded upon activation of the MEN [26]. Consistent with the above result, Clb2 began to accumulate at approximately 60 min after release in both wild-type and *BFA1^4D^* mutant cells, peaked at 90 min, and then declined (Figure 8D and 8E). These results showed that *BFA1^4D^* cells allow timely mitotic exit like wild-type *BFA1*. Together they confirm that phosphorylation of ^452^S, ^453^S, ^454^S, and ^559^S regulates the asymmetric localization of Bfa1 and timely mitotic exit in unperturbed cell cycle.

In addition, the asymmetric presence of Bfa1^4D^ protein on SPBs in G2/M did not affect early mitotic progression (Figure 8). Therefore we suggest that Bfa1 asymmetry is required for timely activation of the MEN but is not necessary for mitotic progression before anaphase in the unperturbed cell cycle.

### Phospho-mimetic *BFA1^4D^* cells delay mitotic exit in response to spindle misalignment

**Figure 9:**
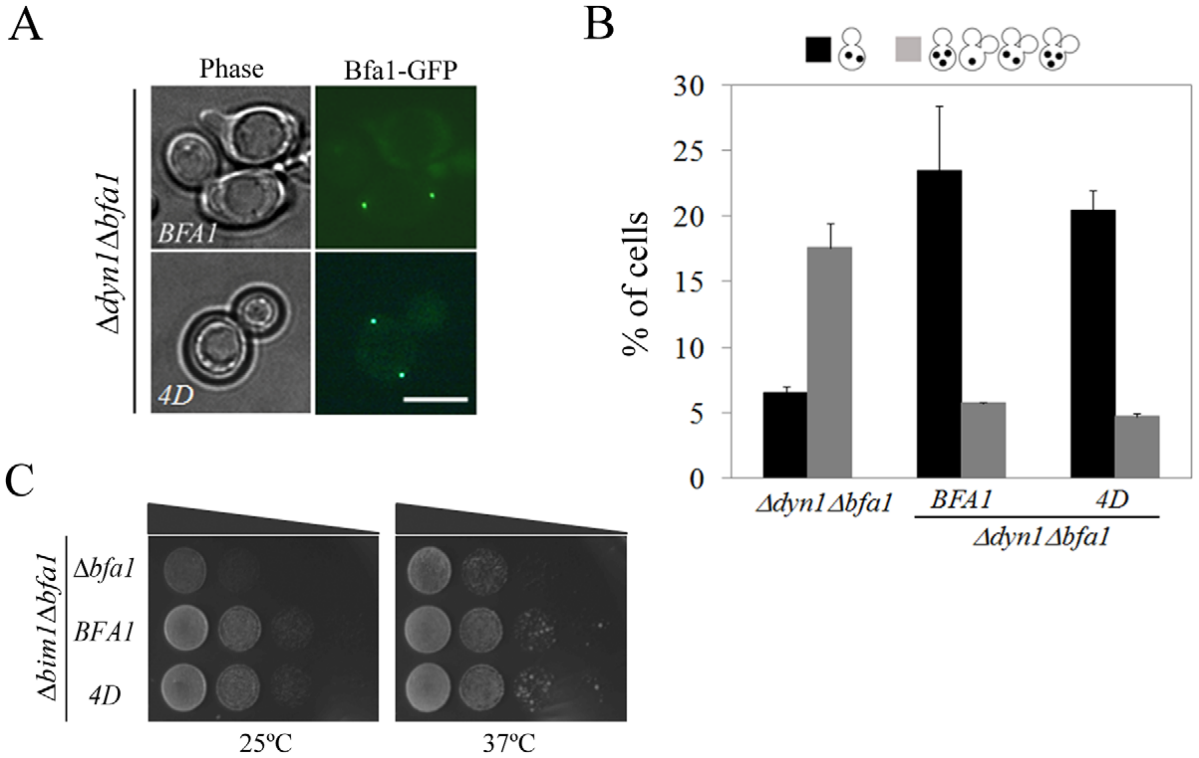
Localization and SPOC activity of Bfa1^4D^ in cells with misaligned spindle. (A) The localization of Bfa1^4D^ in cells with misaligned spindles. *Δdyn1BFA1-GFP* (YSK2103) and *Δdyn1mCherry-TUB1BFA1^4D^-GFP* (YSK2875) cells were synchronized with α-factor and released at 16°C for 24 h. The localization of Bfa1^4D^ in *Δdyn1mCherry-TUB1Δbfa1* mutant cells was observed by fluorescence microcopy. Bar, 5 µm. (B,C) The ability of Bfa1^4D^ mutants to prevent mitotic exit in *Δdyn1Δbfa1* and *Δbim1Δbfa1* cells. (B) *Δdyn1mCherry-TUB1Δbfa1* (YSK2893), *Δdyn1mCherry-TUB1BFA1-GFP* (YSK2894) and *Δdyn1mCherry-TUB1BFA1^4D^-GFP* (YSK2875) cells were synchronized with α-factor at 30°C and released at 16°C for 24 h. Cells with indicated phenotypes were quantified (n = 600) after staining with DAPI. (C) *Δbim1Δbfa1* (YSK1867), *Δbim1BFA1* (YSK2093), and *Δbim1BFA1^4D^* (YSK2898) were serially diluted on YPAD and incubated at either 25°C or 37°C.

Previous studies have suggested that the symmetrical distribution of Bfa1/Bub2 is directly related to the delay of mitotic exit when the spindle is not properly aligned [15], [16]. We therefore asked whether Bfa1^4D^ is able to function in the spindle position checkpoint and whether it is symmetrically localized in cells with misaligned spindles. Proper positioning of the mitotic spindle relies on two independent pathways, one involving the minus-end microtubule motor dynein, the other Bim1, a plus-end microtubule-binding protein [27], [28]. The absence of *DYN1* induces anaphase spindle misalignment in the mother cell and thus triggers the spindle position checkpoint [6], [27]. We first examined the localization of Bfa1^4D^ in *Δdyn1* cells by integrating *pRS304-BFA1^4D^-GFP* into the TRP1 locus of *Δdyn1mCherry-TUB1Δbfa1* cells, as described in Materials and Methods. Surprisingly, Bfa1^4D^ was present on both SPBs in cells with misaligned spindles like wild-type Bfa1 (Figure 9A).

When the anaphase spindle is misaligned in the parent of *Δdyn1* cells, *BFA1* deletion induces improper mitotic exit, as a result of which both multinucleate and anucleate cells accumulate [14], [29]. To assess the spindle position checkpoint functioning of Bfa1^4D^, we monitored multinucleate and anucleate cells in *Δdyn1BFA1^4D^* and compared them with *Δdyn1* cells with the wild-type *BFA1* (*Δdyn1BFA1*) after arrest with α-factor and release at 16°C, when the spindle orientation defect is most pronounced. As shown in Figure 9B, the improper mitotic exit seen in *Δdyn1Δbfa1* cells was significantly decreased in both *Δdyn1BFA1* and *Δdyn1BFA1^4D^* cells; 5.7±0.1% in *Δdyn1BFA1* and 4.7±0.3% in *Δdyn1BFA1^4D^* cells. We also examined the spindle position checkpoint function of phospho-mimetic Bfa1^4D^ in *Δbim1Δbfa1* cells. Consistent with the above result, *BFA1^4D^* rescued the viability of *Δbim1Δbfa1* cells like wild-type *BFA1* (Figure 9C).

These results demonstrate that *BFA1^4D^* cells contain SPOC activity like wild-type *BFA1* cells. The symmetric localization of Bfa1^4D^ is consistent with the SPOC activity of Bfa1^4D^ as well as previous evidence that the symmetrical localization of Bfa1 in cells with misaligned spindles is directly connected to the activation of SPOC [15], [16]. Based on these observations, we suggest that the newly identified Cdc5-dependent phosphorylation residues in Bfa1, ^452^S, ^453^S, ^454^S, and ^559^S, are only important for its asymmetrical localization and the timing of mitotic exit in unperturbed cells.

## Discussion

In budding yeast, Kar9 and dynein preferentially associate with the bud-directed SPB, from which astral microtubules emanate [30], [31]. If these proteins distribute symmetrically to both SPBs, the mitotic spindle does not align properly, showing that SPB asymmetry is essential for mitosis [30], [31]. Konig *et al.* showed that cyclin-dependent kinase 1 (Cdk1) is asymmetrically recruited to the SPB^m^ in early anaphase and negatively regulates MEN activity at the SPB^m^ [32]. Caydasi and Pereira [19] reported that forced targeting of Bfa1 and Bub2 to both SPBs compromised SPOC function and Valerio-Santiago *et al.* demonstrated that control of Tem1 localization is essential for the proper functioning of the MEN and SPOC [13]. Recently, Bertazzi *et al.* showed that Lte1-promoted exclusion of Kin4 from the SPB^d^ is essential for proper mitotic exit [33]. These previous studies mainly focused on the biological function of SPB asymmetry in cells with misaligned spindles. Here, we have demonstrated that Cdc5-dependent phosphorylation of Bfa1 contributes to its asymmetric distribution at SPB^d^, which is required for timely mitotic exit and, therefore, is required for the fidelity of cell division in unperturbed cells without misaligned spindles.

Previously, GAP activity-defective Bub2-Myc was reported to lead to localization of the Bfa1/Bub2 complex to both SPBs throughout the cell cycle. This complex inhibits mitotic exit, but only in mutant backgrounds in which the MEN is partially impaired [17]. However, it was not clear whether the inhibition of mitotic exit was due to lack of asymmetry or to the absence of GAP activity. Therefore, the importance of Bfa1 asymmetry and its specific function in normal cell cycle progression were not well understood. In this study, we identified various asymmetry-defective Bfa1 mutants that persist on both SPBs throughout the cell cycle. In particular, unlike Bub2-Myc, the Bfa1^4A^ mutant stimulated the Tem1 GTPase (Figure 6D), and activated checkpoints for mitotic exit control (Figure S5A–S5C). These results demonstrate that wild-type Bfa1 and Bfa1^4A^ differ only in their localization patterns. Bfa1^4A^ delayed Sic1 accumulation and Cdc14 release by approximately 10 min relative to cells with normal localization of Bfa1 (Figure 7B–7D). On the other hand, the phospho-mimetic Bfa1^4D^ mutant allowed timely mitotic exit like wild-type Bfa1 (Figure 8C–8E). Therefore, we suggest that Bfa1 asymmetry and its disappearance from the SPB^m^ regulate the timing of MEN activation in unperturbed cell division cycles.

However in cells with misaligned spindles, Bfa1^4D^ was located on both SPBs and there was full SPOC activity (Figure 9). We therefore consider that the newly identified Cdc5-dependent phosphorylation residues in Bfa1, ^452^S, ^453^S, ^454^S, and ^559^S, are only important for its asymmetrical localization and the timing of mitotic exit in unperturbed cells. The symmetric localization as well as the SPOC activity of Bfa1^4D^ is consistent with previous studies that showed that the symmetrical localization of Bfa1 in cells with misaligned spindles is directly connected with activation of the SPOC [15], [16]. We speculate that cells override the Cdc5-depedent asymmetric localization of Bfa1 in the presence of a spindle orientation defect. Thus, the previously reported mechanisms that account for the symmetric localization of Bfa1 and the arrest of mitotic exit in response to misaligned spindles may apply to Bfa1^4D^.

It has been suggested that a 10 min delay in the cell division cycle is not biologically significant in controlling the cell division cycle. However, considering that the entire cell cycle of budding yeast is about 90 min and mitosis takes approximately 30 min [34], a 10 min delay is not negligible. In fact, mitotic exit is only delayed by 15 minutes in the presence of constant peak levels of Clb2, which blocks spindle disassembly [35].

Valerio-Santiago *et al.* recently showed that localization of Tem1 to the SPBs is essential for activation of the MEN [13]. As we showed in Figure 5G, Tem1-RFP localized to both SPBs in *BFA1^4A^* cells. We also showed that Bfa1^4A^ binds to Tem1 like wild-type Bfa1 (Figure S6C). These observations suggest that the delay of mitotic exit in *BFA1^4A^* cells is a consequence of disrupting the asymmetric localization of Tem1.

What molecular details underlie Bfa1 asymmetry in unperturbed mitosis? One significant contribution may come from cell polarity determinants [18]. Monje-Casas and Amon reported that the correct interaction of astral microtubules with the bud cortex alters the affinity of Bfa1 for SPBs and affects its asymmetry [18]. Consistent with this observation, Geymonat *et al.* showed that the activity of Lte1 in mitotic regulation depends on its localization to the bud cortex and contributes to the asymmetric localization of Bfa1 to the daughter SPB [12]. How can information in the cortex control the distribution of Bfa1 at SPBs, and how is Bfa1 able to bind to the SPBs with different affinities? When spindles misalign, Kin4 kinase activity and its localization to SPBs are reported to regulate the residence time of Bfa1 at SPBs, as well as SPOC activity [19]. However, if the spindle is correctly aligned, Kin4 begins to associate with the SPB^m^ in mid-anaphase at a time when Bfa1 asymmetry has already been established [36]. In addition, in *Δkin4* cells with proper spindle positioning, Bfa1 has a normal localization pattern [19]. Furthermore, symmetric localization of Bfa1^4A^ is not caused by its defective interaction with Kin4, since Bfa1^4A^ interacted with Kin4 like wild-type Bfa1 (Figure S5D). Thus, other factors must regulate Bfa1 asymmetry, particularly in unperturbed cells. Fraschini *et al.* [17] proposed that the disappearance of Bfa1/Bub2 from the mother-directed SPB requires Bfa1/Bub2 GAP activity. We also observed that various GAP activity-defective Bfa1 mutants persisted at the SPB^m^ during anaphase. However, we showed that Bfa1 asymmetry was not dependent on GAP activity. Because MEN activation requires inhibition of Bfa1 GAP activity and Bfa1 asymmetry, if Bfa1 asymmetry is regulated by its GAP activity, only a decline in GAP activity could promote Bfa1 loss from the SPB^m^. Nevertheless, this is probably not the case, as is shown by the symmetric localization of the GAP-defective Bub2-Myc and Bfa1^DDR2^ proteins. Bfa1 also localized on both SPBs in *BFA1^4A^* cells with normal GAP activity, and in *cdc5-2* cells where Bfa1 GAP activity is expected to be high due to lack of phosphorylation.

Although we have shown in this study that Bfa1 asymmetry is not dependent on its GAP activity, we should consider the possibility that its GAP activity influences its localization indirectly, by affecting its phosphorylation. However, Bfa1^D416A^, which retains approximately 50% of the GAP activity of wild-type Bfa1, is also defective in phosphorylation by Cdc5, like Bfa1 mutants completely lacking GAP activity (Figure 3). Thus, it is unlikely that the GAP activity of Bfa1 influences its localization indirectly by affecting its phosphorylation. It may still be possible that asymmetric localization requires a certain threshold level of Bfa1 GAP activity (which must be higher than the level in Bfa1^D416A^) as a prerequisite for phosphorylation of the four serine residues that we have identified.

Our observations that mutation of the four Cdc5-depedent phosphorylation residues, ^452^S, ^453^S, ^454^S, and ^559^S to Ala in Bfa1^4A^ significantly reduced its phosphorylation by Cdc5, as well as affecting its localization, strongly support the role of these residues in directing asymmetric localization in unperturbed mitosis. This notion was further supported by phospho-mimetic Bfa1^4D^, which was asymmetrically localized to the SPBs in *cdc15-2*-dependent arrested cells (Figure 8A) and even in kinase-defective *cdc5-2* (Figure 8B). However, Bfa1^4D^ were not asymmetrically localized in 100% of the *cdc5-2* cells (Figure 8B) and phosphorylation of Bfa1-D8^11A+4A^ by Cdc5 was only reduced by approximately 25% compared with Bfa1-D8 (Figure 6B). Therefore, we cannot exclude the possibility that Bfa1 contains some additional residue(s) that is/are also phosphorylated by Cdc5 and is/are involved in the asymmetric localization of Bfa1.

Bfa1^4A^ bound to both SPBs of anaphase-arrested *cdc15-2* cells with properly segregated nuclei, whereas the 11 previously identified Cdc5 target sites (Bfa1-11A) had little or no effect on Bfa1 localization. However, the association of Bfa1^4A^ with the two SPBs at anaphase was not as stable as that of wild-type Bfa1 for the SPB^d^. We also found that the Bfa1^DDR2^ mutant formed a stronger association with the SPBs than wild-type Bfa1 (Figure S8). Thus, further characterization of this mutant may help uncover the molecular mechanisms underlying Bfa1 dynamics. One possibility is that all Bfa1 becomes phosphorylated, and phospho-Bfa1 has different affinities for the two SPBs. Alternatively, Cdc5 may differentially phosphorylate Bfa1 at one of the two SPBs. Another possibility raised by Monje-Casas and Amon [18], is that some proteins mediating the association of Bfa1 with SPBs may control the affinity of Bfa1 for the SPBs by introducing various modifications.

When we mapped the phosphosites of Bfa1 by mass spectrometry, phosphorylation of several characterized sites, such as ^7^T and ^424^S, was detected with higher efficiency than phosphorylation of the novel residues we identified as required for Bfa1 asymmetry (Figure S9). It is tempting to speculate that the Cdc5 target sites regulating Bfa1 asymmetry in unperturbed mitosis are phosphorylated with higher fidelity, and/or that other factor(s) are involved in modulating the efficiency of phosphorylation to control Bfa1 dynamics. Due to low phosphorylation efficiency, only ^453^S and ^454^S were detected as phosphor forms by MS. The low phosphorylation efficiency of these residues is consistent with the proposed biological role of their phosphorylation in controlling the timing of mitotic exit during unperturbed cell division cycles. Although phosphorylation of the newly identified phosphosites of Bfa1 by Cdc5 was not very efficient, a similar SSS^934^FL sequence in Claspin had been identified as the target of phosphorylation by Plx1, a frog ortholog of budding yeast Cdc5 [37].

While mapping the Cdc5 target sites, we identified p^150^S and p^180^S, which were reported to be phosphorylated by Kin4 and to prevent further modification of Bfa1 by Cdc5 (10; [38]). Although we cannot exclude the possibility that p^150^S and p^180^S were phosphorylated nonspecifically due to the extremely high Cdc5 kinase activity, the phosphorylation efficiency at these sites was comparable to that of other Cdc5 targets responsible for GAP activity, such as ^7^T and ^424^S (Figure S9). Furthermore, despite the phosphorylation of these sites, other sites were phosphorylated by Cdc5. Thus, we suspect that Bfa1 phosphorylation by Cdc5 and Kin4, and the biological functions of these modifications are far more complicated than we currently understand, and will require further study.

We found that the ^452^S, ^453^S, ^454^S in budding yeast Bfa1 are conserved as ^589^S, ^590^T, ^591^S in its fission yeast homologue byr4 (Figure S11), which is also localized asymmetrically to SPBs in anaphase [39]. It would be interesting to examine whether these conserved residues of byr4 are also phosphorylated by a polo-like kinase, contribute to the asymmetric localization of byr4 and regulate the timing of SIN in fission yeast.

In summary, we have shown that Cdc5-mediated phosphorylation of the newly identified residues on Bfa1 modulates the affinity of Bfa1 for SPBs, and as a consequence contributes to the asymmetric distribution of Bfa1 at anaphase. The asymmetric Bfa1 distribution is required for timely mitotic exit, thus probably ensuring tight coupling of MEN activation and chromosome segregation during normal cell cycle progression. We have also uncovered a novel function of the polo kinase, Cdc5, in the control of mitotic exit. Further studies are needed to identify factors that control the Cdc5-dependent Bfa1 phosphorylation responsible for asymmetric localization in unperturbed mitosis, and how it is overridden in the presence of a misaligned spindle. These studies promise to provide crucial insights into how centrosome asymmetry is generated, and its biological importance in the asymmetric division of eukaryotic cells.

## Materials and Methods

### Yeast strains, culture, and cell cycle synchronization

All yeast cultures and genetic techniques were carried out as described by Kim *et al.* [22]. The *S. cerevisiae* strains used in this study are described in Table S1. Strains were generated by PCR-based methods and verified by PCR, and Southern and western blot analysis [40], [41]. The integrating plasmid, *pRS304-BFA1-GFP*, was linearized with *Eco*RV and integrated into the *TRP1* locus, as described by Kim *et al.* [10]. Bfa1 mutants were constructed by PCR-based site-directed mutagenesis, as described by Kim *et al.* [10]. Cells were synchronized in G1 by adding 10 µg/ml or 50 ng/ml α-factor (Sigma-Aldrich) to *BAR1* or *bar1* cells, respectively, and at S phase with 0.2 M hydroxyurea (Sigma-Aldrich) for 2–3 h. *CDC5* expression was driven by the *GAL* promoter.

### Microscopy and imaging analysis

Fluorescence microscopy was performed essentially as described by Kim *et al.* [22]. Cellular labeling was visualized on an Axioplan2 (Zeiss) microscope with a Zeiss 100× Plan Neofluar oil immersion objective. Images were acquired using an Axiocam CCD (Zeiss) camera and AxioVision software (Zeiss). The fluorescence intensity of GFP and RFP-fused proteins was quantitatively analyzed by confocal microscopy. For confocal images, we used a Nipkov disk-based UltraVIEW RS confocal system (PerkinElmer) equipped with a Nikon microscope (TE2000-PFS). The 100× NA 1.4 oil immersion objective lens was controlled by a piezoelectric z stepper. In each experiment, 10 to 15 z sections were acquired at 0.5 µm steps with 2×2 binning, the same laser power and exposure time, and projected in UltraVIEW RS software (PerkinElmer). Fluorescence intensity of selected regions of interest was quantified using UltraVIEW RS and Image J software (Version 1.38u, NIH), and the background fluorescence was subtracted by placing the same measurement circle in nearby intracellular regions without a Bfa1-GFP or Tem1-RFP signal. Since the fluorescence intensity of Bfa1-GFP generally increased with Spc42-RFP intensity, we normalized the intensity of Bfa1-GFP to Spc42-RFP to precisely measure the amount of Bfa1 associated with the SPB. For time-lapse experiment, a Nipkov disk-based UltraVIEW RS confocal system (PerkinElmer) equipped with a Nikon microscope (TE2000-PFS) was used. Images for cells on agar plugs were taken every 5 min and processed with Adobe Photoshop 7.0. No manipulations were added other than adjustments in brightness and contrast.

### Protein analyses and preparation of recombinant proteins

For phosphatase treatment, TAP-tagged Bfa1 was precipitated with IgG Sepharose beads (Amersham) from total cellular lysates (1 mg in 700 µl modified H-buffer containing 1% NP-40) as described by Kim *et al.* [10] and treated with Calf Intestinal Alkaline Phosphatase (CIP, New England Biolabs) for 30 min at 37°C. For co-immunoprecipitation of TAP-tagged Bfa1^4A^ and 3XHA-tagged Kin4, Kin4 was purified with anti-HA (Roche) followed by protein A-agarose (sigma) and co-precipitates were blotted with peroxidase anti-peroxidase (PAP, Sigma) for Bfa1 or Bfa1^4A^. For western blot analysis, peroxidase anti-peroxidase (PAP, Sigma), monoclonal anti-HA (Roche), monoclonal anti-Myc (Roche), polyclonal anti-GFP (Santa Cruz Biotechnology), Clb2 (produced in the lab), monoclonal anti-α-Tubulin (Sigma), and polyclonal anti-Actin (Santa Cruz Biotechnology) were used. Band intensity was quantified and analyzed using the LAS-3000 image analyzer (Fujifilm) and Image J software (Version 1.38u, NIH).

GST-Cdc5 (*Δ*70N-Cdc5 harboring S165D and T238D) and GST-Cdc5KD (*Δ*70N-Cdc5 harboring an N209A) were expressed in *S. cerevisiae*, as described by Geymonat *et al.* [9]. Tem1, GST-Bub2, MBP-Bfa1, and MBP-D8-Bfa1 were prepared from *E. coli*, as described by Kim *et al.* [10].

### *In vitro* Tem1 GTPase assay and yeast two-hybrid assay

The intrinsic or GAP-stimulated GTPase activity of Tem1 was quantified, as described by Kim *et al.* [10] with an EnzCheck Phosphate Assay Kit (E-6646; Molecular Probes). The amount of γ-P_i_ released from Tem1-GTP was monitored by measuring the absorbance at 360 nm. Yeast two-hybrid assays were performed as previously described Kim *et al.* [22].

### *In vitro* kinase assay

*In vitro* kinase assays were performed, as described by Geymonat *et al.* [9]. For radioactive kinase assays, 100 ng of substrate was mixed with 10–50 ng of either GST-Cdc5 or GST-Cdc5KD in 15 µl kinase buffer (50 mM Tris-Cl, pH 7.5, 10 mM MgCl_2_, 1 mM dithiothreitol) with 50 µM ATP and 0.1 µl γ-[^32^P]ATP (Amersham Biosciences, 370 MBq/ml, 3000 Ci/mmol). After incubation at 30°C for either 15 min for full-length Bfa1 or 30 min for Bfa1-D8, Laemli buffer was added to stop the phosphorylation reaction. γ-[^32^P]-labeling was visualized by autoradiography. To better detect the phosphorylation of Bfa1-D8 by Cdc5, 1–10 µg substrates were incubated with 1–3 µg of either GST-Cdc5 or GST-Cdc5KD in the same kinase buffer with 0.5 mM non-radioactive ATP, separated on 7.5% SDS-PAGE containing 100 µM Phos-tag acrylamide (MANAC Incorporated) [42] and 200 µM MnCl_2_, and stained with Coomassie brilliant blue.

### Determination of Cdc5-dependent phosphorylation by mass spectrometry

After *in vitro* phosphorylation of Bfa1 was performed with purified Cdc5 kinase and the product was digested with trypsin, liquid chromatography was carried out on a Dionex LC Packings nano HPLC system (LC-Packings) coupled to the QSTAR Pulsar ESI-hybrid Q-TOF tandem mass spectrometer (Applied Biosystems), as described in Lee *et al* [43]. The column outlet was coupled directly to the high voltage ESI source (typically 2.3 kV) and peptides eluting from the column were sprayed directly into the orifice of the mass spectrometer. Information-Dependent Acquisition (IDA) mode was performed to acquire MS/MS spectra based on an inclusion mass list and dynamic assessment of relative ion intensity. For MS/MS, a full mass scan range mode was *m/z* = 100–2000 Da. After determining the charge states of an ion on zoom scans, product ion spectra were acquired in MS/MS mode with relative collision energy of 55%. The individual spectra from MS/MS were processed using the Analyst QS software (v1.1, Applied Biosystems) and searched against a limited database containing only the protein of interest, Bfa1, which was performed with mass tolerance 0.1 Da and with a confidence value no less.

## Supporting Information

Figure S1 Verification of the single copy integration and expression levels of Bfa1 mutants. *cdc15-2SPC42-RFP* cells expressing the indicated *BFA1-GFP* mutants (YSK1165, 2545, 2547, 2549, 2551, 2553, and 2557) were analyzed. (A) A genomic Southern blot assessing the single-copy integration of *BFA1* or each *BFA1* mutant. Genomic DNA of indicated cells was digested with *Eco* RI and detected with the 836 bp *Hind* III-*Eco* RI fragment of *BFA1* as a probe, as described by Kim *et al.* [10]. Integration into the *TRP1* locus of *Δbfa1* background cells generated a 3731 bp fragment (arrow) after the genomic DNA was digested with *Eco* RI. (B) The quantification of the expression levels of Bfa1 mutants. In actively growing cells, the expression levels of GFP-tagged version of each Bfa1 derivative were quantified and are plotted relative to actin. Actin was used as an internal loading control.
(TIF)

Figure S2 The function of Bfa1^DDR2^ in mitotic exit. Bfa1^DDR2^ is unable to prevent mitotic exit in mitotic exit-defective cells, such as *Δlte1* (low temperature) and *Δlte1Δste20*, and in response to various checkpoint-activating signals, such as DNA damage, spindle damage, and spindle misorientation. The expression of Bfa1-GFP and Bfa1^DDR2^-GFP was verified by western blot analysis. (A) The ability of Bfa1^DDR2^ to suppress the growth rate of *Δlte1Δbfa1* cells. *Δlte1BFA1* (YSK2052), *Δlte1Δbfa1* (YSK2051), and *Δlte1BFA1^DDR2^* (YSK2218) cells were serially diluted on YPAD and incubated at either 25°C for 2 days or 13°C for 10 days. (B) The growth rate of *Δlte1Δste20BFA1^DDR2^* mutant. *Δlte1Δste20Δbfa1* cells with *pURA3-LTE1* plasmid were transformed with *BFA1* or the *BFA1^DDR2^* mutant. Indicated cells (YSK2063, 2062, and 2237) were serially diluted on either YPAD or YPAD containing 5-FOA and incubated at 25°C for 2–3 days. (C) The ability of Bfa1^DDR2^ to prevent mitotic exit when DNA is damaged. *cdc13-1* (YSK2073), *cdc13-1Δbfa1* (YSK1138), and *cdc13-1BFA1^DDR2^* (YSK2317) were synchronized with α-factor and released into YPAD at 34°C. At each time point, the cells were collected to analyze DNA content by FACS (n = 50,000). (D) The ability of Bfa1^DDR2^ to prevent mitotic exit when spindles are damaged. The indicated cells (YSK2083, 1077, and 2164) were synchronized with α-factor and released into YPAD containing 15 µg/ml nocodazole at 25°C. At each time point, cells with either large buds or new bud formation were scored (n = 200). (E) The ability of Bfa1^DDR2^ to prevent mitotic exit when spindles are improperly positioned. *Δbim1* (YSK2093), *Δbim1Δbfa1* (YSK1867), and *Δbim1BFA1^DDR2^* (YSK2276) were serially diluted on YPAD and incubated at either 25°C or 37°C.
(TIF)

Figure S3 Phosphorylation of GAP activity-defective Bfa1 mutants in cells overexpressing *CDC5*. The indicated cells (YSK2121, 2142, 2143, 2144, 2145, and 2472) were transformed with *pGAL* (vector only) or *pGAL-CDC5*, arrested with 0.2 M hydroxyurea in raffinose medium, and then treated with galactose (t = 0) to induce *CDC5* overexpression. Cells were harvested at each indicated time point. P is a positive control used as in Figure 1A. No Bfa1 phosphorylation was observed (t = 0) in cells arrested in S-phase with hydroxyurea. However, Cdc5 induction was followed by the appearance of phosphorylated forms in *BFA1* and *BFA1^G411E^* cells, seen as the accumulation of slowly migrating bands. In contrast, no slower migrating forms of Bfa1 were detected in other *BFA1* mutants, even after 8 h of *CDC5* overexpression.
(TIF)

Figure S4 Phosphorylation of Bfa1 mutants in cells overexpressing *CDC5*. (A) Phosphorylation of Bfa1-11A, Bfa1^M413I^-11A, Bfa1^D416A^-11A, Bfa1^W422A^-11A, and Bfa1^4A^ -11A in cells overexpressing *CDC5*. Indicated cells (YSK2121, 2147, 2444, 2468, 2470, and 2336) were transformed with *pGAL* or *pGAL-CDC5*, arrested with 0.2 M hydroxyurea in raffinose medium, and then treated with galactose (t = 0) to induce *CDC5* overexpression. Cells were harvested at each indicated time point. P is a positive control used as in Figure 1A. (B) Condition of *BFA1*, *BFA1-11A*, and *BFA1^4A^-11A* cells after Cdc5 was overexpressed in S-phase arrest cells for 10 h in (A). The morphology of *BFA1*, *BFA1-11A*, and *BFA1^4A^-11A* cells were shown to show the viability of these cells, when Cdc5 was overexpressed in S-phase arrest cells for 10 h. Cells without *CDC5* overexpression (t = 0) were shown as controls. Bar, 20 µm.
(TIF)

Figure S5 The ability of the Bfa1^4A^ and Bfa1-11A mutants to prevent mitotic exit in response to checkpoint-activating signals. The GAP activity of Bfa1 mutants for Tem1 was analyzed *in vivo* by the ability to suppress mitotic exit [10]. The expression of GFP-fused Bfa1 mutants was verified by western blot with anti-GFP. Actin was used as a loading control. (A) Spindle damage. The indicated cells (YSK1077, 2083, 2151, 2149, 2435, and 2152) were synchronized with α-factor and released into YPAD containing 15 µg/ml nocodazole at 25°C. At each time point, cells with either large buds or new bud formation were scored (n = 200). (B) DNA damage. Indicated cells (YSK1138, 2073, 2314, 2315, 2484, and 2313) were grown at 25°C, synchronized with α-factor, and released into fresh YPAD at 34°C. At each time point, the percentage of cells with either large buds or new buds was determined (n = 200). (C) Spindle orientation defects. The indicated cells (YSK1129, 2103, 2485, 2486, 2487, and 2488) were synchronized with α-factor at 30°C and released into YPAD at 16°C for 24 h. Cells were stained with DAPI, and cells with indicated phenotypes were quantified (n = 200). The average of three independent counts is plotted with standard deviations. (D) The physical interaction of wild-type Bfa1 and Bfa1^4A^ with Kin4. *KIN4-3HAΔbfa1* (YSK2910), *KIN4-3HABFA1-TAP* (YSK2911) and *KIN4-3HABFA1^4A^-TAP* (YSK2912) cells were grown at 25°C to mid-log phase and harvested. Crude extracts were prepared, and Kin4 was purified with anti-HA followed by protein A-agarose as described in Materials and Methods. Bfa1 and Kin4 were detected with PAP and anti-HA, respectively.
(TIF)

Figure S6 The physical interaction of asymmetry-defective Bfa1^4A^ and GAP activity-defective Bfa1 mutants with Cdc5, Tem1, and Bub2 by yeast two-hybrid assays. Cdc5, Tem1 and Bub2 were fused to the DNA activation domain in pJG4-5, and Bfa1 and each Bfa1 mutant were fused to the DNA binding domain in pGilda. The yeast strain EGY48 was co-transformed with these constructs and the reporter plasmid pSH18-34. Western blots show that the similar amount of proteins was included in each assay. (A, C and D) The interaction of wild-type Bfa1 and Bfa1^4A^ mutant with (A) cdc5, (C) Tem1 or (D) Bub2 was measured quantitatively. (B) The interaction of wild-type Bfa1 and GAP activity-defective Bfa1 mutants with Cdc5 was measured quantitatively.
(TIF)

Figure S7 The ability of Bfa1^4D^ to suppress the growth of *cdc5-2Δbfa1* cells. *cdc5-2Δbfa1* (YSK2526), *cdc5-2BFA1* (YSK2606), and *cdc5-2BFA1^4D^* (YSK2907) were grown to mid-log phase, then serially diluted on YPAD and incubated at either 25°C or 37°C.
(TIF)

Figure S8 The fluorescence intensity of Bfa1^DDR2^-GFP at SPBs. The box plots compare the fluorescence intensities of Bfa1-GFP at SPBs. *cdc15-2SPC42-RFPBFA1-GFP* (YSK2545) and *cdc15-2SPC42-RFPBFA1^DDR2^-GFP* (YSK2557) cells were released for 3 h at 35°C from α-factor synchronization. GFP fluorescence signals were analyzed as described in Materials and Methods (n = 30 for Bfa1 and 41 for Bfa1^DDR2^). The line inside the box indicates the median. D, GFP signal at the daughter SPB. M, GFP signal at the mother SPB. Note that this experiment was accomplished in parallel with Figure 5F, and the wild-type Bfa1 control is the same in both cases.
(TIF)

Figure S9 Analysis of Cdc5-dependent phosphorylation by mass spectrometry. In-gel tryptic digests of the *in vitro* phosphorylated Bfa1 with purified Cdc5 kinase were analyzed by LC-MS/MS. The Cdc5 phosphorylation efficiency at known Bfa1 phospho-residues and the novel residues responsible for asymmetry that we identified in this study is plotted. Among the eight peptides with ^7^T residue, peptide including p^7^T (phosphorylated ^7^T) was detected twice with 25% efficiency (2/8). ^150^S, ^180^S, ^424^S, and ^454^S were detected in phospho-form with 25% (3/12), 25% (5/20), 25% (5/20), and 16.7% (2/12) efficiency respectively.
(TIF)

Figure S10 The Cdc5-dependent phosphorylation of ^150^S and ^180^S. The MS/MS spectra of doubly charged mass/charge (m/z) = 437.186^2+^ were used to search against a limited database containing only the protein of interest, Bfa1, and corresponds to (A) a Bfa1 peptide ^150^pSMMELKPK^157^ with a phosphorylated ^150^Ser and (B) ^180^pSMPNLALVNPAIR^192^ with a phosphorylated ^180^Ser. The b and y ions detected are marked on the peaks. The mass of 98 on the peaks was derived from neutral losses (−97.9769 Da) of phosphoric acid from the precursor ion. Peaks are seen for ions which have water (−18 Da) denoted y° and b°.
(TIF)

Figure S11 Sequence alignment Bfa1-D8 with its fission yeast homologue byr4. (A) ^452^S, ^453^S, and ^454^S residues of Bfa1 that are necessary for asymmetric localization of Bfa1 to the SPB^d^ are conserved in byr4. The amino acid sequence similarity between Bfa1 and byr4 was analyzed by BLAST program (NCBI). Conserved residues are shown in red.
(TIF)

Table S1 Yeast strains used in this study. Yeast strains constructed and used in this study were listed in the following table.
(DOCX)
