# Supplementary figures and images for: Cdc5-Dependent Asymmetric Localization of Bfa1 Fine-Tunes Timely Mitotic Exit
Source: PLoS Genet. 2012 Jan 12;8(1):e1002450. doi: 10.1371/journal.pgen.1002450 (PMC3257293; doi:10.1371/journal.pgen.1002450)

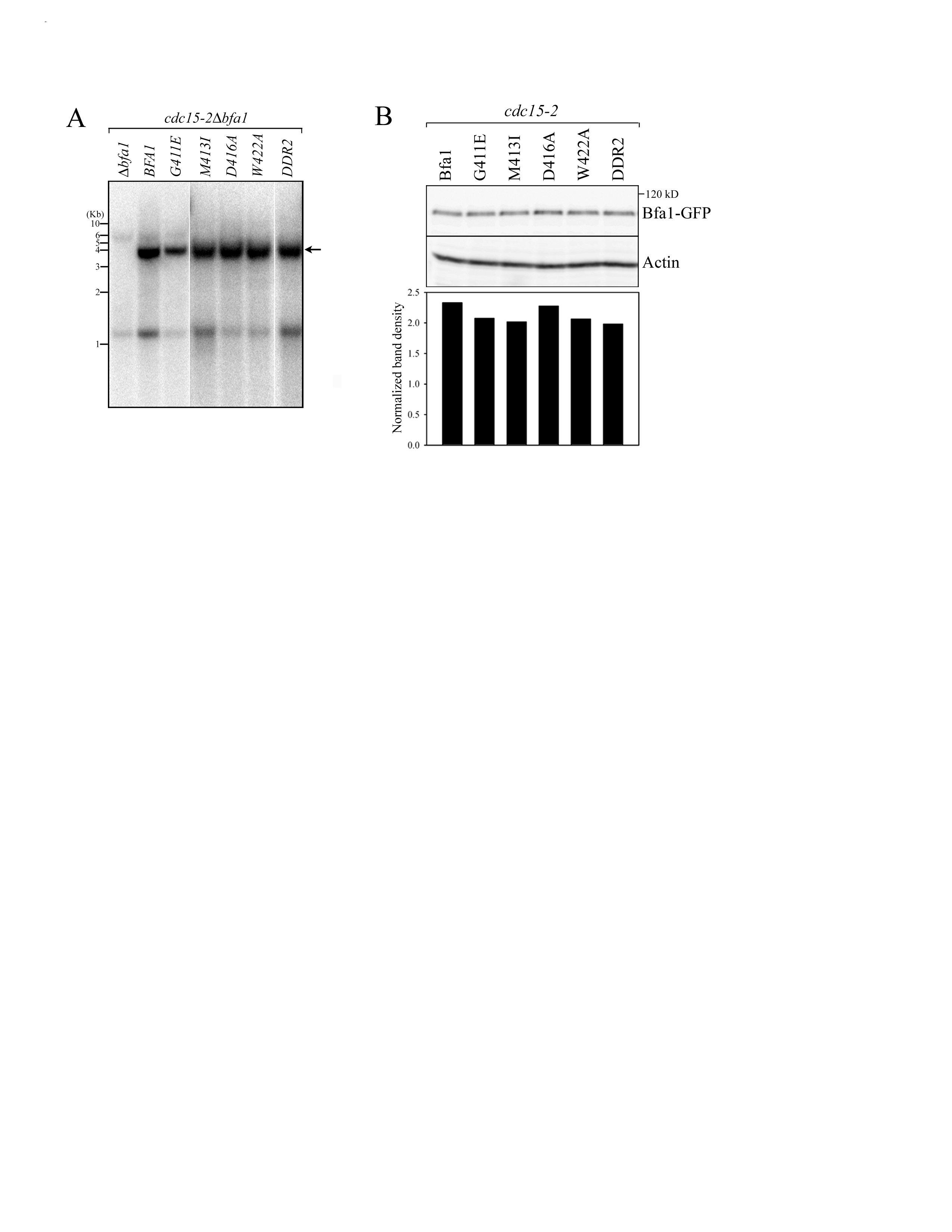

Supplement: Figure S1 — Verification of the single copy integration and expression levels of Bfa1 mutants. cdc15-2SPC42-RFP cells expressing the indicated BFA1-GFP mutants (YSK1165, 2545, 2547, 2549, 2551, 2553, and 2557) were analyzed. (A) A genomic Southern blot assessing the single-copy integration of BFA1 or each BFA1 mutant. Genomic DNA of indicated cells was digested with Eco RI and detected with the 836 bp Hind III-Eco RI fragment of BFA1 as a probe, as described by Kim et al. [10]. Integration into the TRP1 locus of Δbfa1 background cells generated a 3731 bp fragment (arrow) after the genomic DNA was digested with Eco RI. (B) The quantification of the expression levels of Bfa1 mutants. In actively growing cells, the expression levels of GFP-tagged version of each Bfa1 derivative were quantified and are plotted relative to actin. Actin was used as an internal loading control. (TIF) [file pgen.1002450.s001.tif]

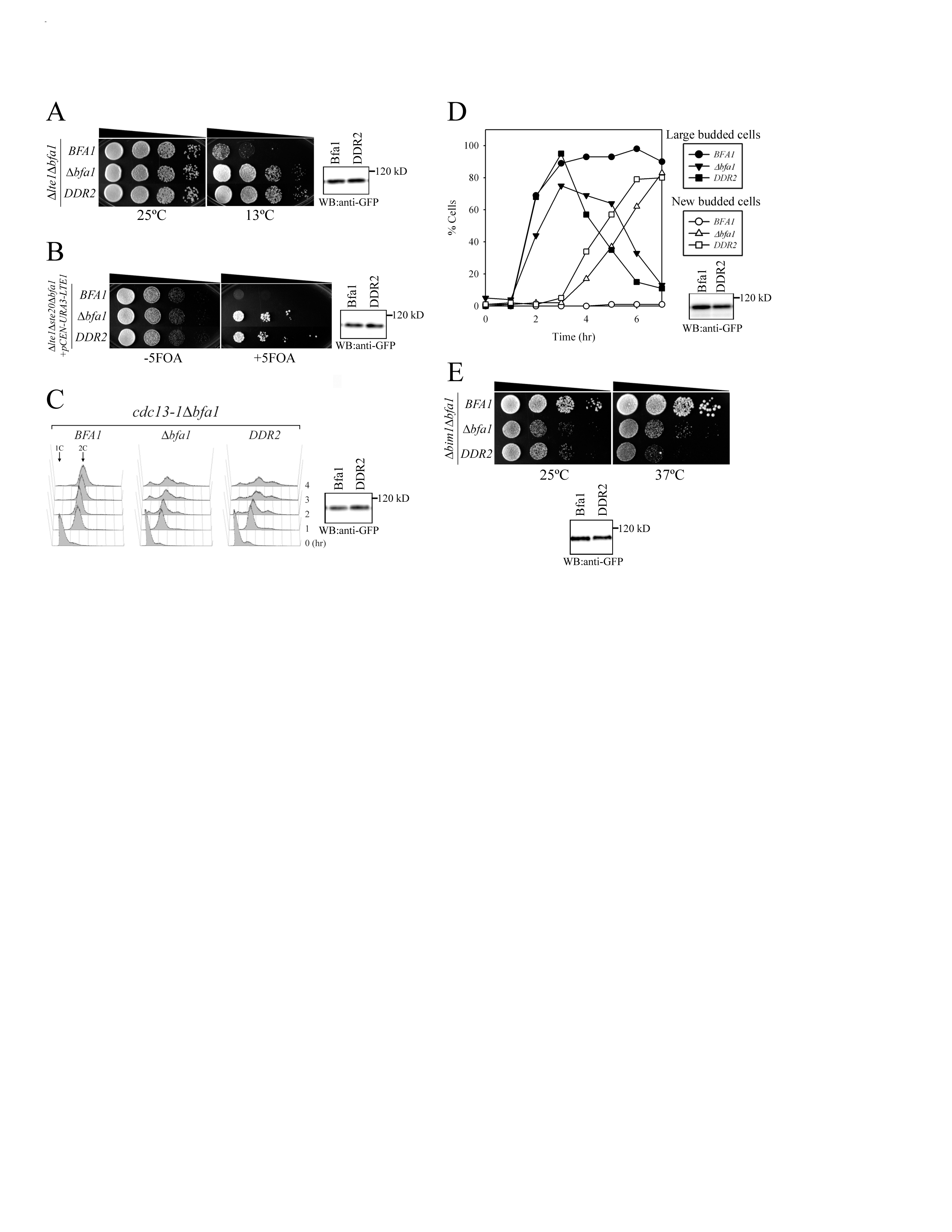

Supplement: Figure S2 — The function of Bfa1DDR2 in mitotic exit. Bfa1DDR2 is unable to prevent mitotic exit in mitotic exit-defective cells, such as Δlte1 (low temperature) and Δlte1Δste20, and in response to various checkpoint-activating signals, such as DNA damage, spindle damage, and spindle misorientation. The expression of Bfa1-GFP and Bfa1DDR2-GFP was verified by western blot analysis. (A) The ability of Bfa1DDR2 to suppress the growth rate of Δlte1Δbfa1 cells. Δlte1BFA1 (YSK2052), Δlte1Δbfa1 (YSK2051), and Δlte1BFA1DDR2 (YSK2218) cells were serially diluted on YPAD and incubated at either 25°C for 2 days or 13°C for 10 days. (B) The growth rate of Δlte1Δste20BFA1DDR2 mutant. Δlte1Δste20Δbfa1 cells with pURA3-LTE1 plasmid were transformed with BFA1 or the BFA1DDR2 mutant. Indicated cells (YSK2063, 2062, and 2237) were serially diluted on either YPAD or YPAD containing 5-FOA and incubated at 25°C for 2–3 days. (C) The ability of Bfa1DDR2 to prevent mitotic exit when DNA is damaged. cdc13-1 (YSK2073), cdc13-1Δbfa1 (YSK1138), and cdc13-1BFA1DDR2 (YSK2317) were synchronized with α-factor and released into YPAD at 34°C. At each time point, the cells were collected to analyze DNA content by FACS (n = 50,000). (D) The ability of Bfa1DDR2 to prevent mitotic exit when spindles are damaged. The indicated cells (YSK2083, 1077, and 2164) were synchronized with α-factor and released into YPAD containing 15 µg/ml nocodazole at 25°C. At each time point, cells with either large buds or new bud formation were scored (n = 200). (E) The ability of Bfa1DDR2 to prevent mitotic exit when spindles are improperly positioned. Δbim1 (YSK2093), Δbim1Δbfa1 (YSK1867), and Δbim1BFA1DDR2 (YSK2276) were serially diluted on YPAD and incubated at either 25°C or 37°C. (TIF) [file pgen.1002450.s002.tif]

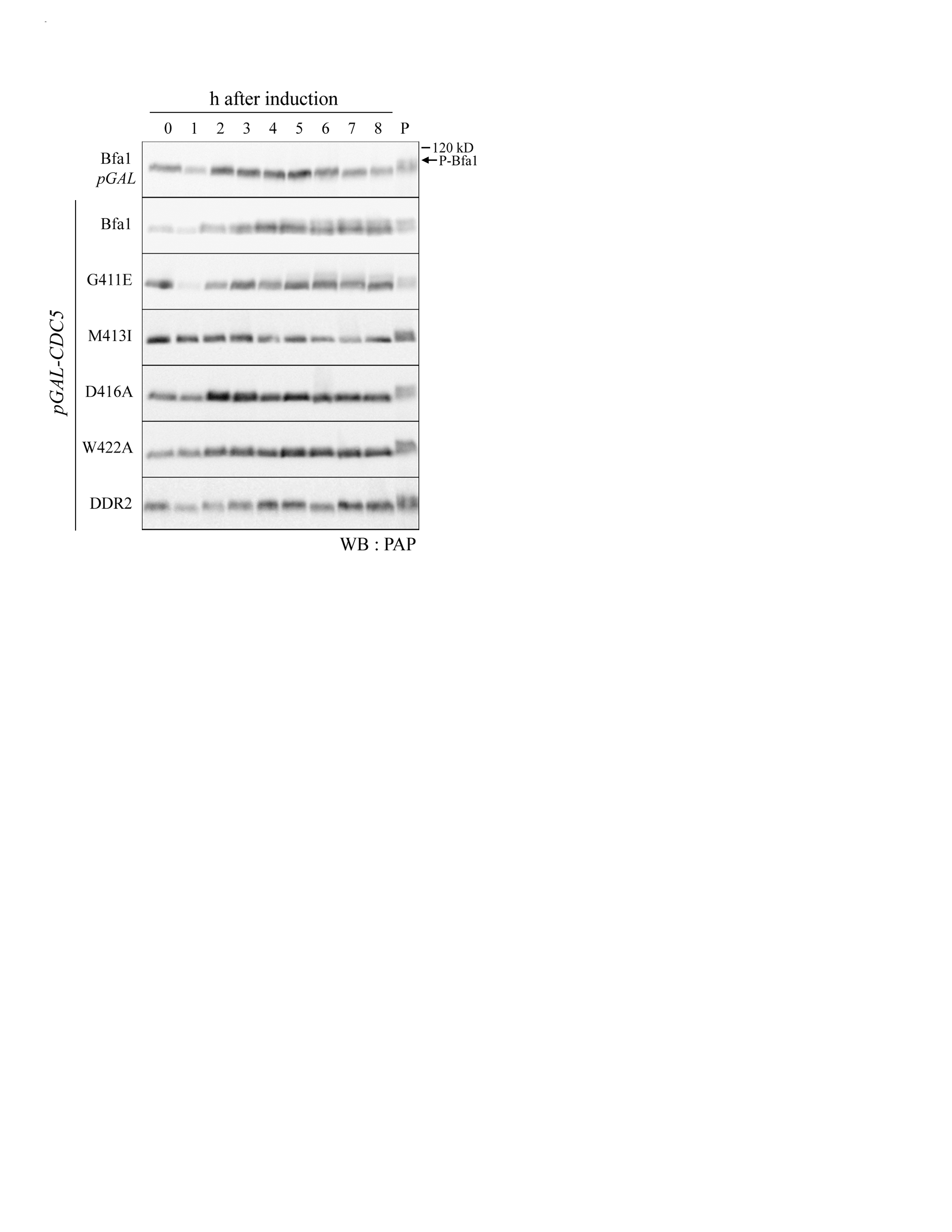

Supplement: Figure S3 — Phosphorylation of GAP activity-defective Bfa1 mutants in cells overexpressing CDC5. The indicated cells (YSK2121, 2142, 2143, 2144, 2145, and 2472) were transformed with pGAL (vector only) or pGAL-CDC5, arrested with 0.2 M hydroxyurea in raffinose medium, and then treated with galactose (t = 0) to induce CDC5 overexpression. Cells were harvested at each indicated time point. P is a positive control used as in Figure 1A. No Bfa1 phosphorylation was observed (t = 0) in cells arrested in S-phase with hydroxyurea. However, Cdc5 induction was followed by the appearance of phosphorylated forms in BFA1 and BFA1G411E cells, seen as the accumulation of slowly migrating bands. In contrast, no slower migrating forms of Bfa1 were detected in other BFA1 mutants, even after 8 h of CDC5 overexpression. (TIF) [file pgen.1002450.s003.tif]

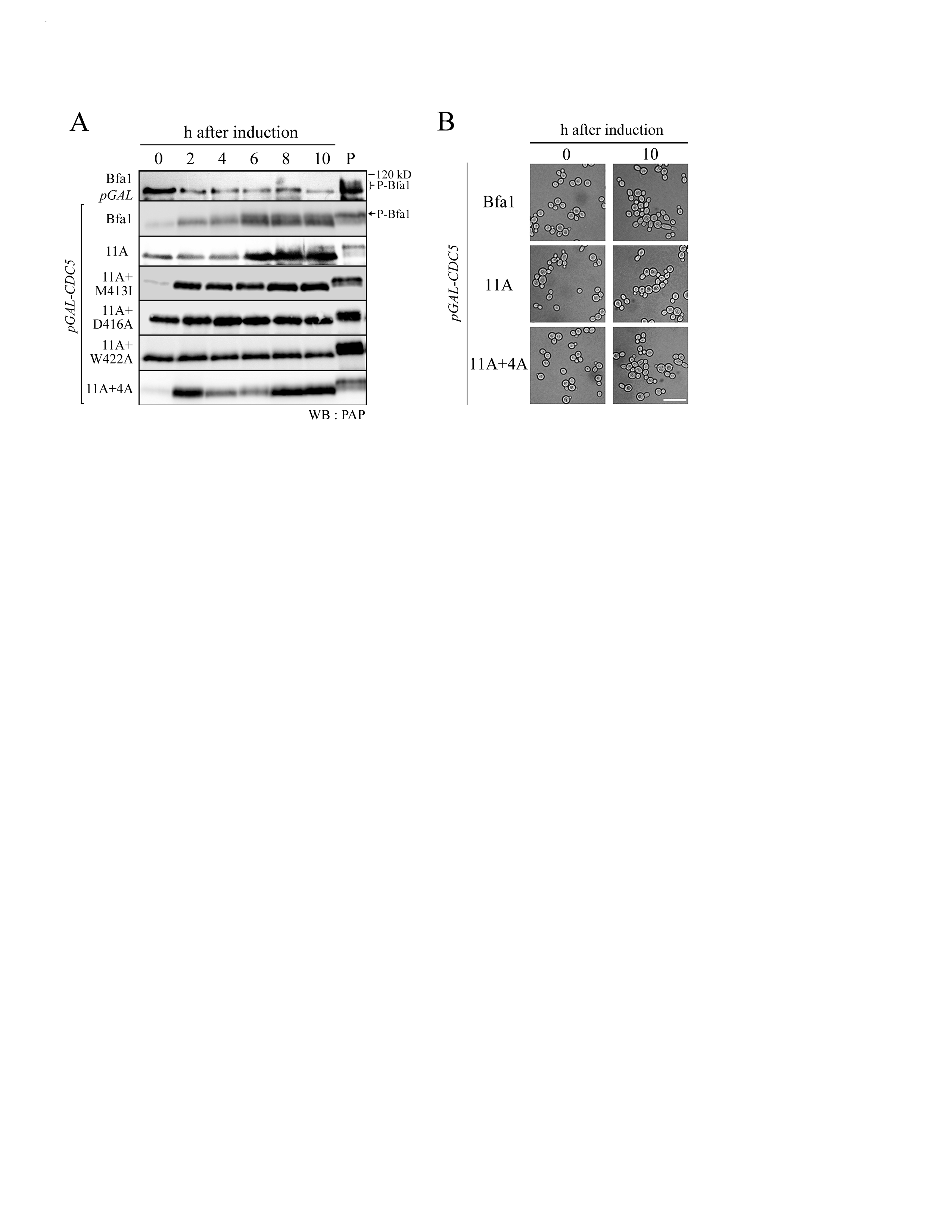

Supplement: Figure S4 — Phosphorylation of Bfa1 mutants in cells overexpressing CDC5. (A) Phosphorylation of Bfa1-11A, Bfa1M413I-11A, Bfa1D416A-11A, Bfa1W422A-11A, and Bfa14A -11A in cells overexpressing CDC5. Indicated cells (YSK2121, 2147, 2444, 2468, 2470, and 2336) were transformed with pGAL or pGAL-CDC5, arrested with 0.2 M hydroxyurea in raffinose medium, and then treated with galactose (t = 0) to induce CDC5 overexpression. Cells were harvested at each indicated time point. P is a positive control used as in Figure 1A. (B) Condition of BFA1, BFA1-11A, and BFA14A-11A cells after Cdc5 was overexpressed in S-phase arrest cells for 10 h in (A). The morphology of BFA1, BFA1-11A, and BFA14A-11A cells were shown to show the viability of these cells, when Cdc5 was overexpressed in S-phase arrest cells for 10 h. Cells without CDC5 overexpression (t = 0) were shown as controls. Bar, 20 µm. (TIF) [file pgen.1002450.s004.tif]

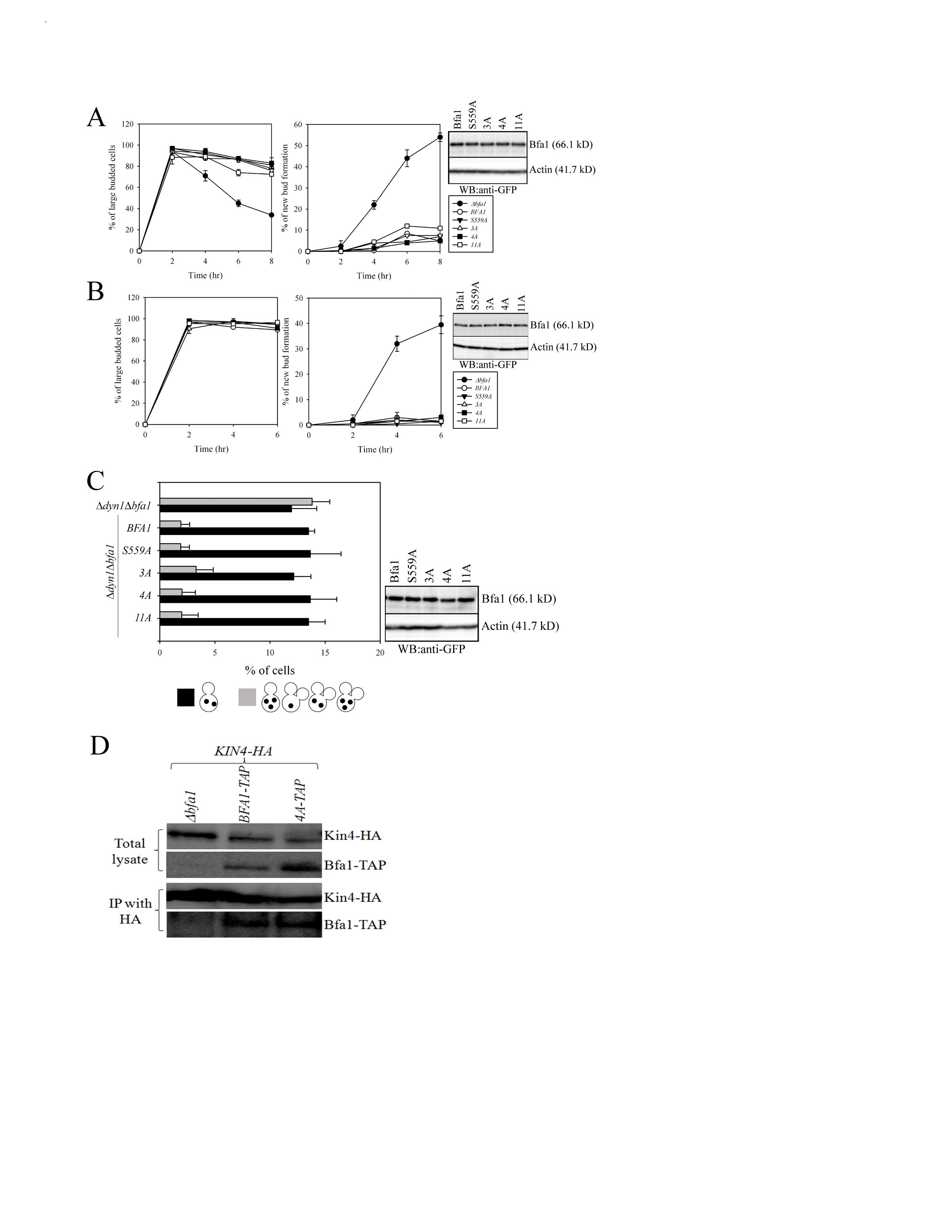

Supplement: Figure S5 — The ability of the Bfa14A and Bfa1-11A mutants to prevent mitotic exit in response to checkpoint-activating signals. The GAP activity of Bfa1 mutants for Tem1 was analyzed in vivo by the ability to suppress mitotic exit [10]. The expression of GFP-fused Bfa1 mutants was verified by western blot with anti-GFP. Actin was used as a loading control. (A) Spindle damage. The indicated cells (YSK1077, 2083, 2151, 2149, 2435, and 2152) were synchronized with α-factor and released into YPAD containing 15 µg/ml nocodazole at 25°C. At each time point, cells with either large buds or new bud formation were scored (n = 200). (B) DNA damage. Indicated cells (YSK1138, 2073, 2314, 2315, 2484, and 2313) were grown at 25°C, synchronized with α-factor, and released into fresh YPAD at 34°C. At each time point, the percentage of cells with either large buds or new buds was determined (n = 200). (C) Spindle orientation defects. The indicated cells (YSK1129, 2103, 2485, 2486, 2487, and 2488) were synchronized with α-factor at 30°C and released into YPAD at 16°C for 24 h. Cells were stained with DAPI, and cells with indicated phenotypes were quantified (n = 200). The average of three independent counts is plotted with standard deviations. (D) The physical interaction of wild-type Bfa1 and Bfa14A with Kin4. KIN4-3HAΔbfa1 (YSK2910), KIN4-3HABFA1-TAP (YSK2911) and KIN4-3HABFA14A-TAP (YSK2912) cells were grown at 25°C to mid-log phase and harvested. Crude extracts were prepared, and Kin4 was purified with anti-HA followed by protein A-agarose as described in Materials and Methods. Bfa1 and Kin4 were detected with PAP and anti-HA, respectively. (TIF) [file pgen.1002450.s005.tif]

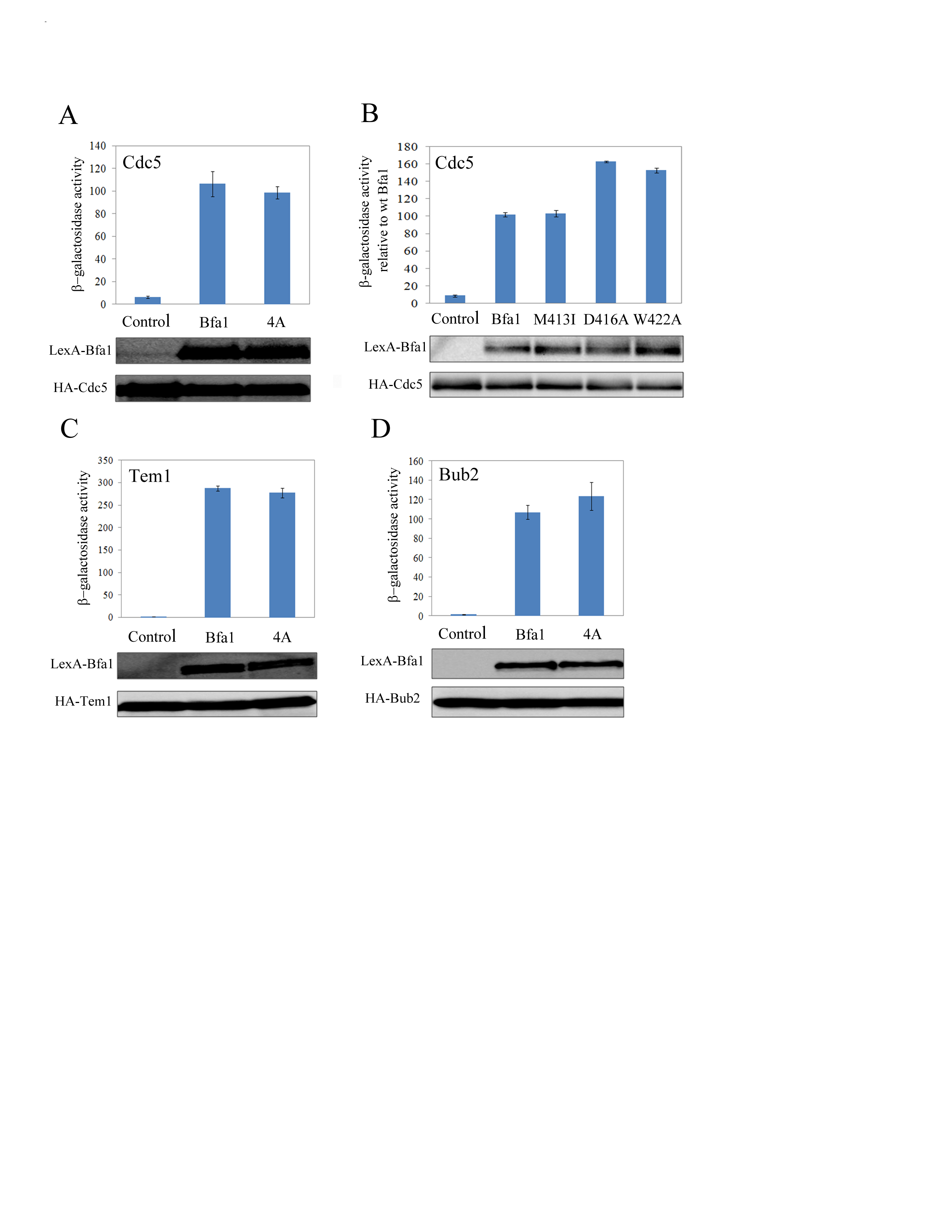

Supplement: Figure S6 — The physical interaction of asymmetry-defective Bfa14A and GAP activity-defective Bfa1 mutants with Cdc5, Tem1, and Bub2 by yeast two-hybrid assays. Cdc5, Tem1 and Bub2 were fused to the DNA activation domain in pJG4-5, and Bfa1 and each Bfa1 mutant were fused to the DNA binding domain in pGilda. The yeast strain EGY48 was co-transformed with these constructs and the reporter plasmid pSH18-34. Western blots show that the similar amount of proteins was included in each assay. (A, C and D) The interaction of wild-type Bfa1 and Bfa14A mutant with (A) cdc5, (C) Tem1 or (D) Bub2 was measured quantitatively. (B) The interaction of wild-type Bfa1 and GAP activity-defective Bfa1 mutants with Cdc5 was measured quantitatively. (TIF) [file pgen.1002450.s006.tif]

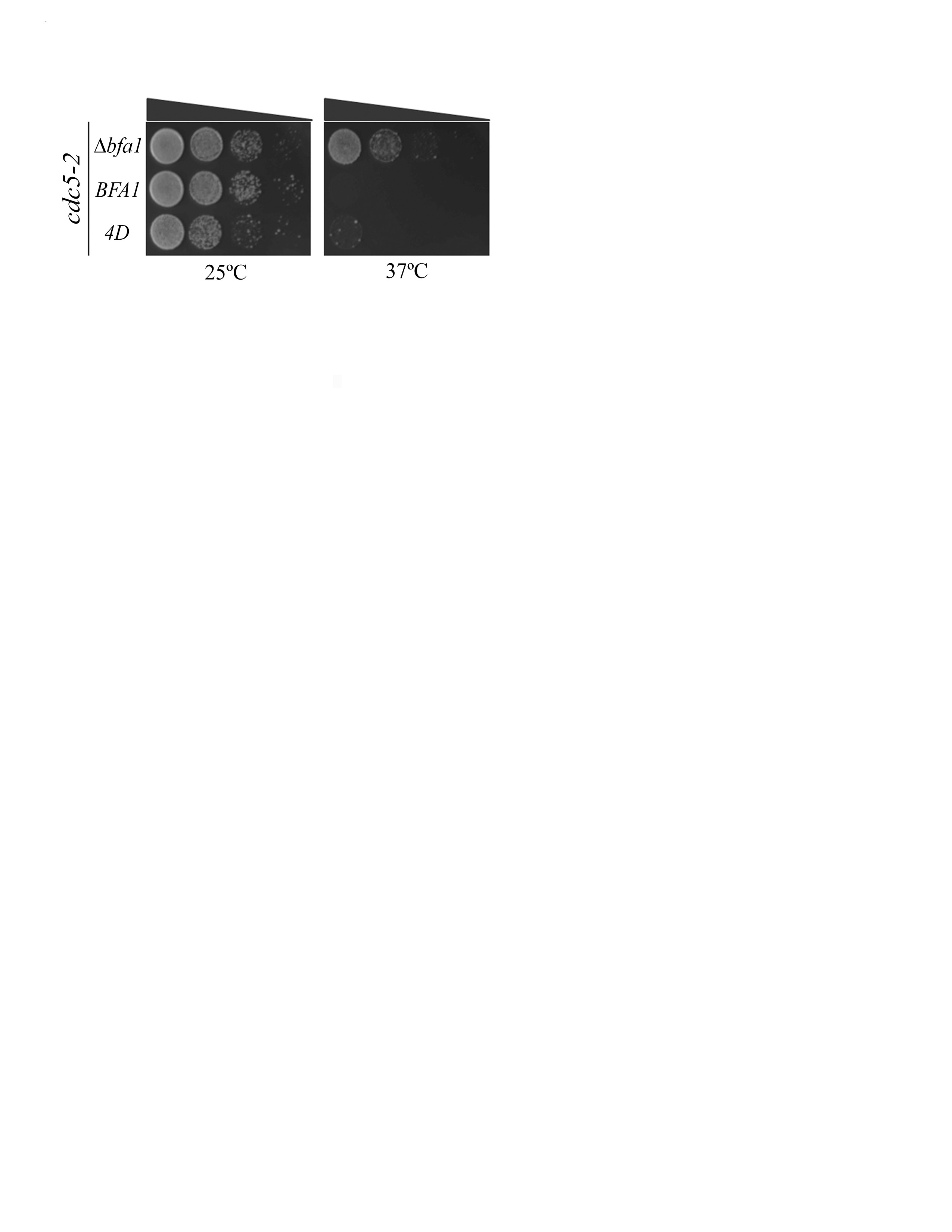

Supplement: Figure S7 — The ability of Bfa14D to suppress the growth of cdc5-2Δbfa1 cells. cdc5-2Δbfa1 (YSK2526), cdc5-2BFA1 (YSK2606), and cdc5-2BFA14D (YSK2907) were grown to mid-log phase, then serially diluted on YPAD and incubated at either 25°C or 37°C. (TIF) [file pgen.1002450.s007.tif]

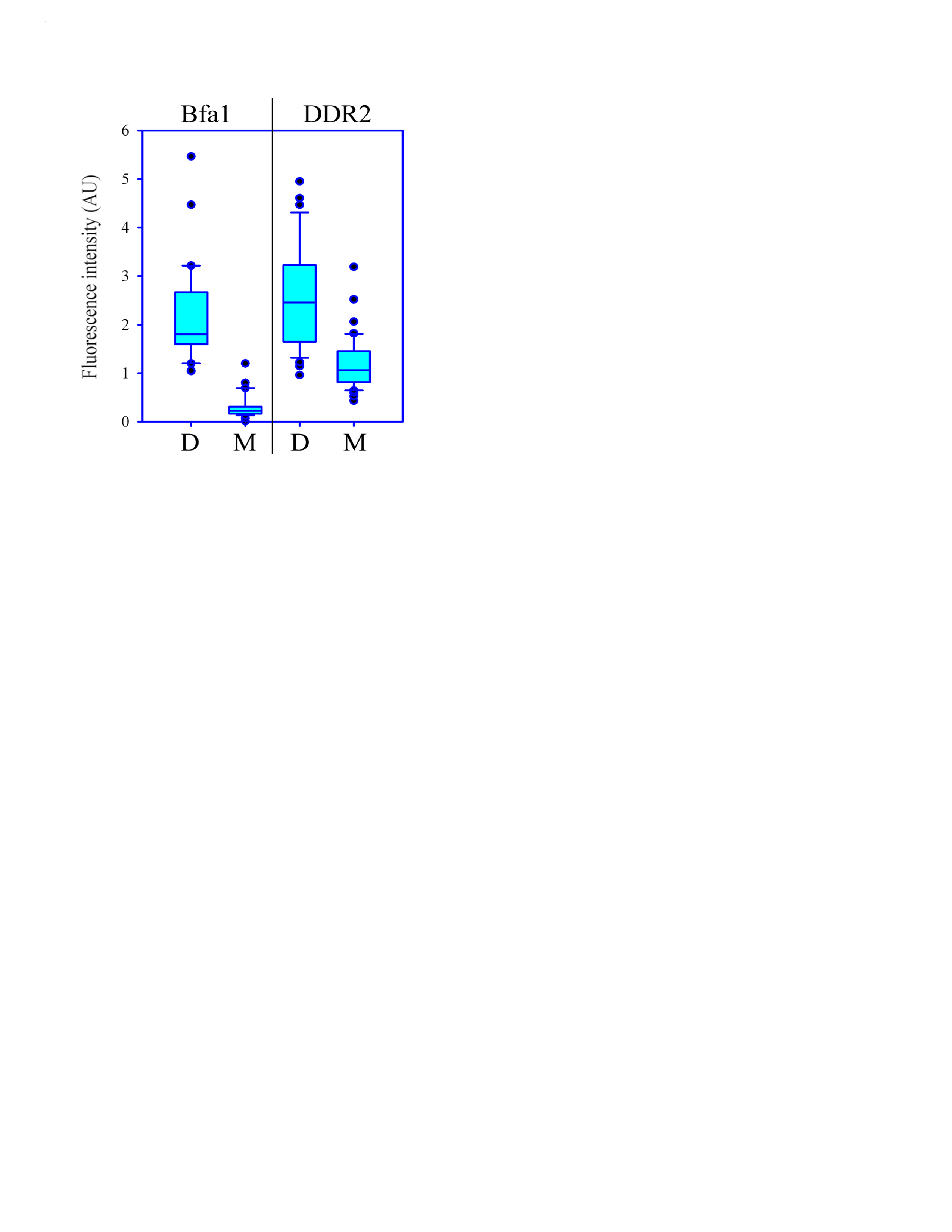

Supplement: Figure S8 — The fluorescence intensity of Bfa1DDR2-GFP at SPBs. The box plots compare the fluorescence intensities of Bfa1-GFP at SPBs. cdc15-2SPC42-RFPBFA1-GFP (YSK2545) and cdc15-2SPC42-RFPBFA1DDR2-GFP (YSK2557) cells were released for 3 h at 35°C from α-factor synchronization. GFP fluorescence signals were analyzed as described in Materials and Methods (n = 30 for Bfa1 and 41 for Bfa1DDR2). The line inside the box indicates the median. D, GFP signal at the daughter SPB. M, GFP signal at the mother SPB. Note that this experiment was accomplished in parallel with Figure 5F, and the wild-type Bfa1 control is the same in both cases. (TIF) [file pgen.1002450.s008.tif]

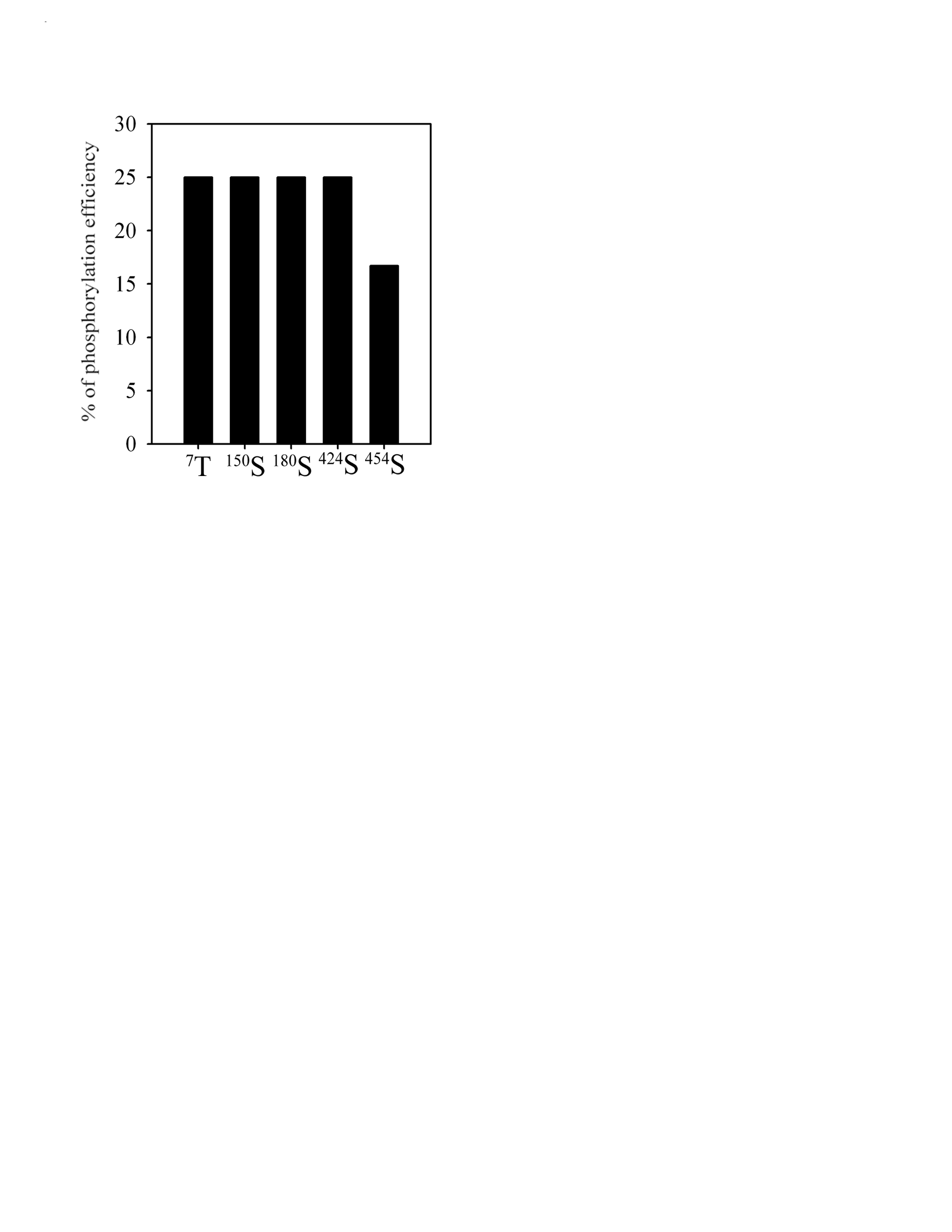

Supplement: Figure S9 — Analysis of Cdc5-dependent phosphorylation by mass spectrometry. In-gel tryptic digests of the in vitro phosphorylated Bfa1 with purified Cdc5 kinase were analyzed by LC-MS/MS. The Cdc5 phosphorylation efficiency at known Bfa1 phospho-residues and the novel residues responsible for asymmetry that we identified in this study is plotted. Among the eight peptides with 7T residue, peptide including p7T (phosphorylated 7T) was detected twice with 25% efficiency (2/8). 150S, 180S, 424S, and 454S were detected in phospho-form with 25% (3/12), 25% (5/20), 25% (5/20), and 16.7% (2/12) efficiency respectively. (TIF) [file pgen.1002450.s009.tif]

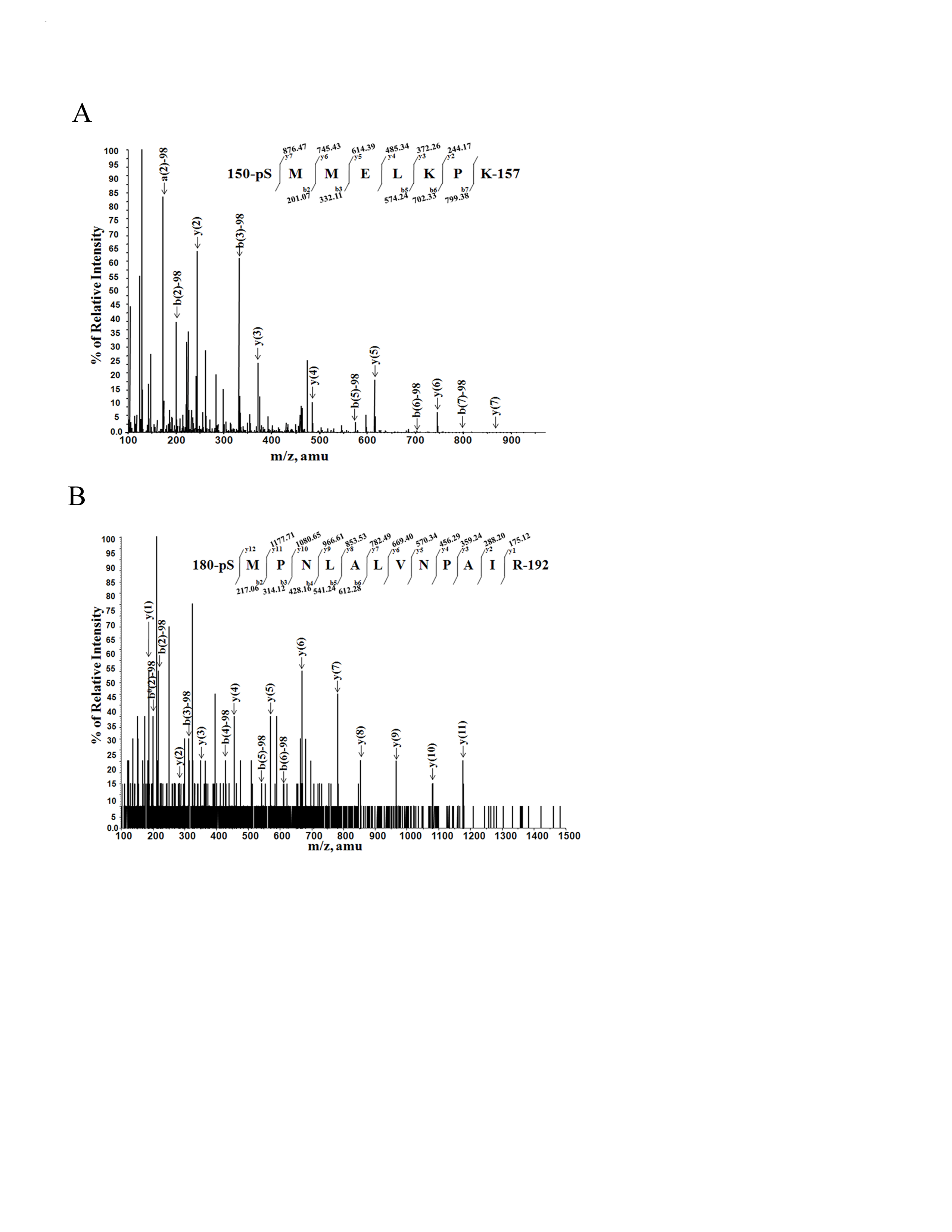

Supplement: Figure S10 — The Cdc5-dependent phosphorylation of 150S and 180S. The MS/MS spectra of doubly charged mass/charge (m/z) = 437.1862+ were used to search against a limited database containing only the protein of interest, Bfa1, and corresponds to (A) a Bfa1 peptide 150pSMMELKPK157 with a phosphorylated 150Ser and (B) 180pSMPNLALVNPAIR192 with a phosphorylated 180Ser. The b and y ions detected are marked on the peaks. The mass of 98 on the peaks was derived from neutral losses (−97.9769 Da) of phosphoric acid from the precursor ion. Peaks are seen for ions which have water (−18 Da) denoted y° and b°. (TIF) [file pgen.1002450.s010.tif]

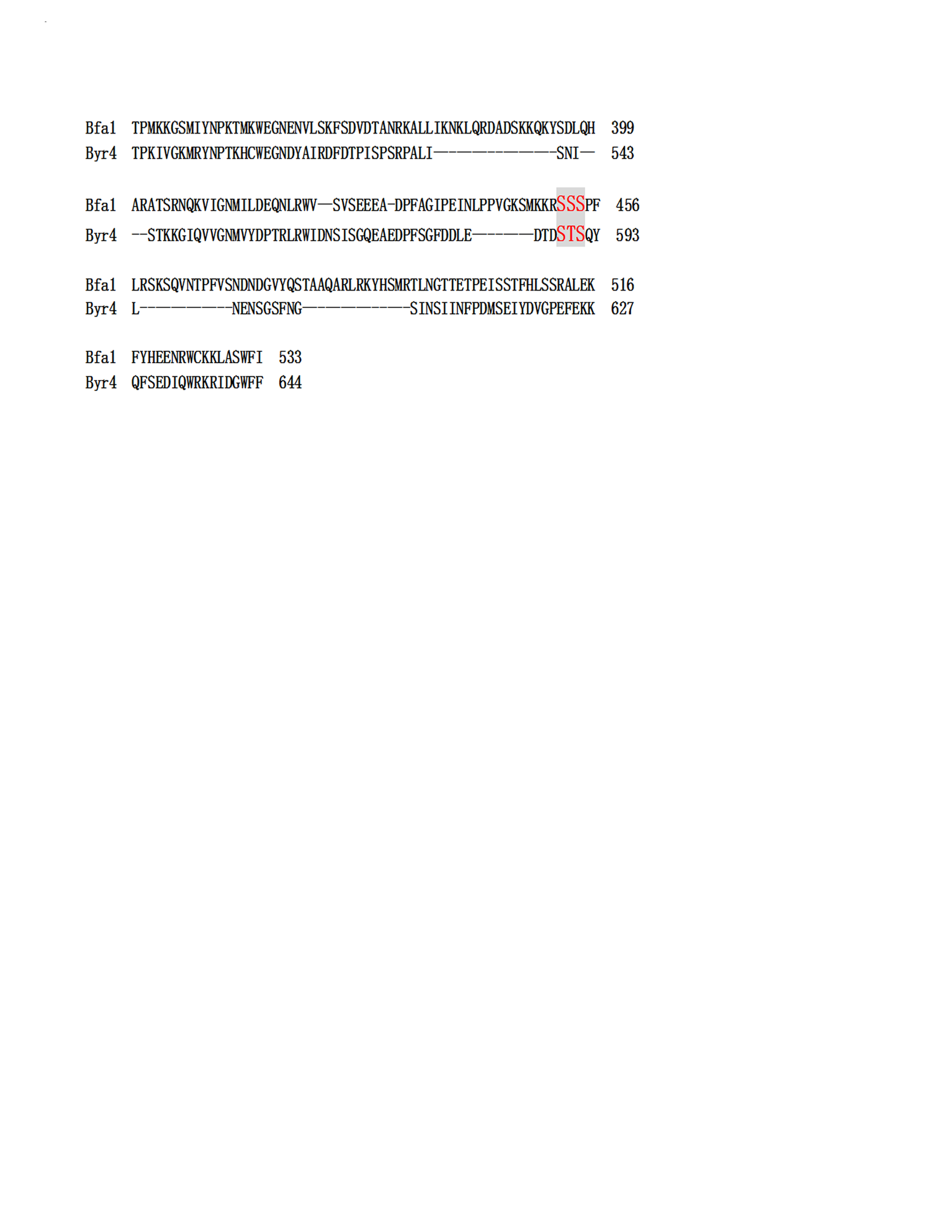

Supplement: Figure S11 — Sequence alignment Bfa1-D8 with its fission yeast homologue byr4. (A) 452S, 453S, and 454S residues of Bfa1 that are necessary for asymmetric localization of Bfa1 to the SPBd are conserved in byr4. The amino acid sequence similarity between Bfa1 and byr4 was analyzed by BLAST program (NCBI). Conserved residues are shown in red. (TIF) [file pgen.1002450.s011.tif]
