# Supplementary material for: Cdc5-Dependent Asymmetric Localization of Bfa1 Fine-Tunes Timely Mitotic Exit
Source: PLoS Genet. 2012 Jan 12;8(1):e1002450. doi: 10.1371/journal.pgen.1002450 (PMC3257293; doi:10.1371/journal.pgen.1002450)
Supplement: Table S1 — Yeast strains used in this study. Yeast strains constructed and used in this study were listed in the following table. (DOCX) [file pgen.1002450.s012.docx]

**Table S1.** Yeast strains used in this study

| **Name** | **Genotype** | **Source*** |
| --- | --- | --- |
| W303a | *MATa* *ade2-1 ura3-1 trp1-1 leu2-3,112 his3-11,15 can1-100* | S. Elledge |
| EGY48 | *MATa LexAop6-LEU2 ura3 his3 trp1 GAL+* | Clontech |
| YSK1077 | W303a except *as bar1 Δbfa1::KAN^R^* |  |
| YSK1122 | W303a except as *cdc5-2::URA3 Δbfa1::his5^+^ [pRS304-BFA1-TAP]* |  |
| YSK1129 | W303a except as *bar1 Δdyn1::LEU2 Δbfa1::KAN^R^* |  |
| YSK1138 | W303a except as *cdc13-1 Δbfa1::HIS3* |  |
| YSK1153 | W303a except as *cdc15-2 Δbar1::LEU2 Δbfa1::his5^+^ [pRS304-BFA1-TAP]* |  |
| YSK1165 | W303a except as *cdc15-2 Δbfa1::his5^+^ SPC42-RFP:KAN^R^* |  |
| YSK1867 | W303a except as *Δbim1::KAN^R^ Δbfa1::HIS3* |  |
| YSK2051 | W303a except as *Δlte1::LEU2 Δbfa1::KAN^R^* |  |
| YSK2052 | W303a except as *Δlte1::LEU2 Δbfa1::KAN^R^ [pRS304-BFA1-GFP]* |  |
| YSK2062 | W303a except as *Δlte1::LEU2 Δbfa1::KAN^R^ Δste20::hphNT1 [pURA3-LTE1]* |  |
| YSK2063 | W303a except as *Δlte1::LEU2 Δbfa1::KAN^R^ Δste20::hphNT1 [pURA3-LTE1] [pRS304-BFA1-GFP]]* |  |
| YSK2073 | W303a except as *cdc13-1 Δbfa1::HIS3 [pRS304-BFA1-GFP]* |  |
| YSK2083 | W303a except as *bar1 Δbfa1::KAN^R^ [pRS304-BFA1-GFP]* |  |
| YSK2093 | W303a except as *Δbim1::KAN^R^ Δbfa1::HIS3 [pRS304-BFA1-GFP]* |  |
| YSK2103 | W303a except as *bar1 Δdyn1::LEU2 Δbfa1::KAN^R^ [pRS304-BFA1-GFP]* |  |
| YSK2121 | W303a except as *bar1 Δbfa1::KAN^R^ [pRS304-BFA1-TAP]* |  |
| YSK2142 | W303a except as *bar1 Δbfa1::KAN^R^ [pRS304-BFA1^G411E^-TAP]* |  |
| YSK2143 | W303a except as *bar1 Δbfa1::KAN^R^ [pRS304-BFA1^M413I^-TAP]* |  |
| YSK2144 | W303a except as *bar1 Δbfa1::KAN^R^ [pRS304-BFA1^D416A^-TAP]* |  |
| YSK2145 | W303a except as *bar1 Δbfa1::KAN^R^ [pRS304-BFA1^W422A^-TAP]* |  |
| YSK2147 | W303a except as *bar1 Δbfa1::KAN^R^ [pRS304-BFA1-11A-TAP]* |  |
| YSK2149 | W303a except as *bar1 Δbfa1::KAN^R^ [pRS304-BFA^3A^-GFP]* |  |
| YSK2151 | W303a except as *bar1 Δbfa1::KAN^R^ [pRS304-BFA1^S559A^-GFP]* |  |
| YSK2152 | W303a except as *bar1 Δbfa1::KAN^R^ [pRS304-BFA1-11A-GFP]* |  |
| YSK2164 | W303a except as *bar1 Δbfa1::KAN^R^ [pRS304-BFA1^DDR2^-GFP]* |  |
| YSK2172 | W303a except as *cdc15-2 Δbar1::LEU2 Δbfa1::his5^+^ [pRS304-BFA1^G411E^-TAP]* |  |
| YSK2173 | W303a except as *cdc15-2 Δbar1::LEU2 Δbfa1::his5^+^ [pRS304-BFA1^M413I^-TAP]* |  |
| YSK2174 | W303a except as *cdc15-2 Δbar1::LEU2 Δbfa1::his5^+^ [pRS304-BFA1^D416A^-TAP]* |  |
| YSK2175 | W303a except as *cdc15-2 Δbar1::LEU2 Δbfa1::his5^+^ [pRS304-BFA1^W422A^-TAP]* |  |
| YSK2177 | W303a except as *cdc15-2 Δbar1::LEU2 Δbfa1::his5^+^ [pRS304-BFA1-11A-TAP]* |  |
| YSK2178 | W303a except as *cdc15-2 Δbar1::LEU2 Δbfa1::his5^+^ [pRS304-BFA1^M413I^-11A-TAP]* |  |
| YSK2179 | W303a except as *cdc15-2 Δbar1::LEU2 Δbfa1::his5^+^ [pRS304-BFA1^D416A^-11A-TAP]* |  |
| YSK2180 | W303a except as *cdc15-2 Δbar1::LEU2 Δbfa1::his5^+^ [pRS304-BFA1^W422A^-11A-TAP]* |  |
| YSK2189 | W303a except as *cdc15-2 Δbar1::LEU2 Δbfa1::his5^+^ [pRS304-BFA1^M413I^-11A-GFP]* |  |
| YSK2190 | W303a except as *cdc15-2 Δbar1::LEU2 Δbfa1::his5^+^ [pRS304-BFA1^D416A^-11A-GFP]* |  |
| YSK2191 | W303a except as *cdc15-2 Δbar1::LEU2 Δbfa1::his5^+^ [pRS304-BFA1^W422A^-11A-GFP]* |  |
| YSK2192 | W303a except as *cdc15-2 Δbar1::LEU2 Δbfa1::his5^+^ [pRS304-BFA1^3A^- GFP]* |  |
| YSK2193 | W303a except as *cdc15-2 Δbar1::LEU2 Δbfa1::his5^+^ [pRS304-BFA1^S453A^- GFP]* |  |
| YSK2194 | W303a except as *cdc15-2 Δbar1::LEU2 Δbfa1::his5^+^ [pRS304-BFA1^S559A^- GFP]* |  |
| YSK2195 | W303a except as *cdc15-2 Δbar1::LEU2 Δbfa1::his5^+^ [pRS304-BFA1-D8-GFP]* |  |
| YSK2196 | W303a except as *cdc15-2 Δbar1::LEU2 Δbfa1::his5^+^ [pRS304-BFA1-D8^M413I^-GFP]* |  |
| YSK2197 | W303a except as *cdc15-2 Δbar1::LEU2 Δbfa1::his5^+^ [pRS304-BFA1-D8^D416A^-GFP]* |  |
| YSK2198 | W303a except as *cdc15-2 Δbar1::LEU2 Δbfa1::his5^+^ [pRS304-BFA1-D8^W422A^-GFP]* |  |
| YSK2202 | W303a except as *bar1 Δbfa1::KAN^R^ PDS1-9myc-hphNT1 SIC1-3HA-his5^+^ [pRS304-BFA1-GFP]* |  |
| YSK2204 | W303a except as *bar1 Δbfa1::KAN^R^ PDS1-9myc-hphNT1 SIC1-3HA-his5^+^ [pRS304-BFA1^M413I^-GFP]* |  |
| YSK2205 | W303a except as *bar1 Δbfa1::KAN^R^ PDS1-9myc-hphNT1 SIC1-3HA-his5^+^ [pRS304-BFA1^D416A^-GFP]* |  |
| YSK2206 | W303a except as *bar1 Δbfa1::KAN^R^ PDS1-9myc-hphNT1 SIC1-3HA-his5^+^ [pRS304-BFA1^W422A^-GFP]* |  |
| YSK2218 | W303a except as *Δlte1::LEU2 Δbfa1::KAN^R^ [pRS304-BFA1^DDR2^-GFP]* |  |
| YSK2237 | W303a except as *Δlte1::LEU2 Δbfa1::KAN^R^ Δste20::hphNT1 [pURA3-LTE1] [pRS304-BFA1^DDR2^-GFP]]* |  |
| YSK2238 | W303a except as *cdc5-1 Δbfa1::his5^+^ [pRS304-BFA1-TAP]* |  |
| YSK2276 | W303a except as *Δbim1::KAN^R^ Δbfa1::HIS3 [pRS304-BFA1^DDR2^-GFP]* |  |
| YSK2313 | W303a except as *cdc13-1 Δbfa1::HIS3 [pRS304-BFA1-11A-GFP]* |  |
| YSK2314 | W303a except as *cdc13-1 Δbfa1::HIS3 [pRS304-BFA1^S559A^-GFP]* |  |
| YSK2315 | W303a except as *cdc13-1 Δbfa1::HIS3 [pRS304-BFA1^3A^-GFP]* |  |
| YSK2317 | W303a except as *cdc13-1 Δbfa1::HIS3 [pRS304-BFA1^DDR2^-GFP]* |  |
| YSK2330 | W303a except as *cdc15-2 Δbar1::LEU2 Δbfa1::his5^+^ [pRS304-BFA1^4A^- GFP]* |  |
| YSK2332 | W303a except as *cdc15-2 Δbar1::LEU2 Δbfa1::his5^+^ [pRS304-BFA1^S452A^- GFP]* |  |
| YSK2334 | W303a except as *cdc15-2 Δbar1::LEU2 Δbfa1::his5^+^ [pRS304-BFA1^S454A^- GFP]* |  |
| YSK2336 | W303a except as *bar1 Δbfa1::KAN^R^ [pRS304-BFA1^4A^-11A-TAP]* |  |
| YSK2338 | W303a except as *bar1 Δbfa1::KAN^R^ PDS1-9myc-hphNT1 SIC1-3HA-his5^+^ [pRS304-BFA1^4A^-GFP]* |  |
| YSK2435 | W303a except as *bar1 Δbfa1::KAN^R^ [pRS304-BFA1^4A^-GFP]* |  |
| YSK2438 | W303a except as *cdc5-1 Δbfa1::his5^+^ SPC42-RFP:KAN^R^ [pRS304-BFA1-GFP]* |  |
| YSK2442 | W303a except as *cdc5-2:URA3 Δbfa1::his5^+^ SPC42-RFP:KAN^R^ [pRS304-BFA1-GFP]* |  |
| YSK2444 | W303a except as *bar1 Δbfa1::KAN^R^ [pRS304-BFA1^M413I^-11A-TAP]* |  |
| YSK2468 | W303a except as *bar1 Δbfa1::KAN^R^ [pRS304-BFA1^D416A^-11A-TAP]* |  |
| YSK2470 | W303a except as *bar1 Δbfa1::KAN^R^ [pRS304-BFA1^W422A^-11A-TAP]* |  |
| YSK2472 | W303a except as *bar1 Δbfa1::KAN^R^ [pRS304-BFA1^DDR2^-TAP]* |  |
| YSK2485 | W303a except as *bar1 Δdyn1::LEU2 Δbfa1::KAN^R^ [pRS304-BFA1^S559A^-GFP]* |  |
| YSK2484 | W303a except as *cdc13-1 Δbfa1::HIS3 [pRS304-BFA1^4A^-GFP]* |  |
| YSK2486 | W303a except as *bar1 Δdyn1::LEU2 Δbfa1::KAN^R^ [pRS304-BFA1^3A^-GFP]* |  |
| YSK2487 | W303a except as *bar1 Δdyn1::LEU2 Δbfa1::KAN^R^ [pRS304-BFA1^4A^-GFP]* |  |
| YSK2488 | W303a except as *bar1 Δdyn1::LEU2 Δbfa1::KAN^R^ [pRS304-BFA1-11A-GFP]* |  |
| YSK2545 | W303a except as *cdc15-2 Δbfa1::his5^+^ SPC42-RFP:KAN^R^ [pRS304-BFA1-GFP]* |  |
| YSK2547 | W303a except as *cdc15-2 Δbfa1::his5^+^ SPC42-RFP:KAN^R^ [pRS304-BFA1^G411E^-GFP]* |  |
| YSK2549 | W303a except as *cdc15-2 Δbfa1::his5^+^ SPC42-RFP:KAN^R^ [pRS304-BFA1^M413I^-GFP]* |  |
| YSK2551 | W303a except as *cdc15-2 Δbfa1::his5^+^ SPC42-RFP:KAN^R^ [pRS304-BFA1^D416A^-GFP]* |  |
| YSK2553 | W303a except as *cdc15-2 Δbfa1::his5^+^ SPC42-RFP:KAN^R^ [pRS304-BFA1^W422A^-GFP]* |  |
| YSK2555 | W303a except as *cdc15-2 Δbfa1::his5^+^ SPC42-RFP:KAN^R^ [pRS304-BFA1-11A-GFP]* |  |
| YSK2557 | W303a except as *cdc15-2 Δbfa1::his5^+^ SPC42-RFP:KAN^R^ [pRS304-BFA1^DDR2^-GFP]* |  |
| YSK2561 | W303a except as *cdc15-2 Δbfa1::his5^+^ SPC42-RFP:KAN^R^ [pRS304-BFA1^4A^-GFP]* |  |
| YSK2617 | W303a except as *cdc15-2 Δbar1::LEU2 Δbfa1::his5^+^ [pRS304-BFA1^DDR2^-TAP]* |  |
| YSK2776 | W303a except as *cdc15-2 Δbfa1:: hphNT1 TEM1-RFP:KAN^R^ [pRS304-BFA1-GFP]* |  |
| YSK2758 | W303a except as *cdc15-2 Δbfa1::hphNT1 TEM1-RFP:KAN^R^ [pRS304-BFA1^4A^-GFP]* |  |
| YSK2858 | W303a except as *cdc15-2 Δbfa1:: his5^+^ SPC42-RFP:KAN^R^ [pRS304-BFA1^4D^-GFP]* |  |
| YSK2854 | W303a except as *CDC14-GFP::TRP1 SPC42-RFP:KAN^R^ Δbfa1::HIS3 [pRS306-BFA1-TAP]* |  |
| YSK2855 | W303a except as *CDC14-GFP::TRP1 SPC42-RFP:KAN^R^ Δbfa1::HIS3 [pRS306-BFA1^4A^-TAP]* |  |
| YSK2898 | W303a except as *Δbim1::KAN^R^ Δbfa1::HIS3 [pRS304-BFA1^4D^-GFP]* |  |
| YSK2910 | W303a except as *bar1 Δbfa1::KAN^R^ KIN4-3HA-hphNT1* |  |
| YSK2911 | W303a except as *bar1 Δbfa1::KAN^R^ KIN4-3HA-hphNT1 [pRS304-BFA1-TAP]* |  |
| YSK2912 | W303a except as *bar1 Δbfa1::KAN^R^ KIN4-3HA-hphNT1 [pRS304-BFA1^4A^-TAP]* |  |
| YSK2526 | W303a except as *cdc5-2::URA3 Δbfa1::hphNT1* |  |
| YSK2606 | W303a except as *cdc5-2::URA3 Δbfa1:: hphNT1 [pRS304-BFA1-GFP]* |  |
| YSK2907 | W303a except as *cdc5-2::URA3 Δbfa1:: hphNT1 [pRS304-BFA1^4D^-GFP]* |  |
| YSK2893 | W303a except as *bar1 Δdyn1::LEU2 Δbfa1::KAN^R^ pRS306-mCherry-TUB1* |  |
| YSK2894 | W303a except as *bar1 Δdyn1::LEU2 Δbfa1::KAN^R^ pRS306-mCherry-TUB1 [pRS304-BFA1-GFP]* |  |
| YSK2875 | W303a except as *bar1 Δdyn1::LEU2 Δbfa1::KAN^R^ pRS306-mCherry-TUB1 [pRS304-BFA1^4D^-GFP]* |  |
|  |  |  |

*All strains for which no source is indicated were constructed in this study.
